# Bulk and single-cell transcriptome analyses of islet tissue unravel gene signatures associated with pyroptosis and immune infiltration in type 2 diabetes

**DOI:** 10.3389/fendo.2023.1132194

**Published:** 2023-03-09

**Authors:** Yaxian Song, Chen He, Yan Jiang, Mengshi Yang, Zhao Xu, Lingyan Yuan, Wenhua Zhang, Yushan Xu

**Affiliations:** ^1^ Department of Endocrinology, Yunnan Province Clinical Medical Center for Endocrine and Metabolic Disease, The First Affiliated Hospital of Kunming Medical University, Kunming, China; ^2^ Department of Geriatric Medicine, The First Affiliated Hospital of Kunming Medical University, Kunming, China

**Keywords:** differentially expressed gene, type 2 diabetes, pyroptosis, immune infiltration, single-cell RNA

## Abstract

**Introduction:**

Type 2 diabetes (T2D) is a common chronic heterogeneous metabolic disorder. However, the roles of pyroptosis and infiltrating immune cells in islet dysfunction of patients with T2D have yet to be explored. In this study, we aimed to explore potential crucial genes and pathways associated with pyroptosis and immune infiltration in T2D.

**Methods:**

To achieve this, we performed a conjoint analysis of three bulk RNA-seq datasets of islets to identify T2D-related differentially expressed genes (DEGs). After grouping the islet samples according to their ESTIMATE immune scores, we identified immune- and T2D-related DEGs. A clinical prediction model based on pyroptosis-related genes for T2D was constructed. Weighted gene co-expression network analysis was performed to identify genes positively correlated with pyroptosis-related pathways. A protein–protein interaction network was established to identify pyroptosis-related hub genes. We constructed miRNA and transcriptional networks based on the pyroptosis-related hub genes and performed functional analyses. Single-cell RNA-seq (scRNA-seq) was conducted using the GSE153885 dataset. Dimensionality was reduced using principal component analysis and t-distributed statistical neighbor embedding, and cells were clustered using Seurat. Different cell types were subjected to differential gene expression analysis and gene set enrichment analysis (GSEA). Cell–cell communication and pseudotime trajectory analyses were conducted using the samples from patients with T2D.

**Results:**

We identified 17 pyroptosis-related hub genes. We determined the abundance of 13 immune cell types in the merged matrix and found that these cell types were correlated with the 17 pyroptosis-related hub genes. Analysis of the scRNA-seq dataset of 1892 islet samples from patients with T2D and controls revealed 11 clusters. INS and IAPP were determined to be pyroptosis-related and candidate hub genes among the 11 clusters. GSEA of the 11 clusters demonstrated that the myc, G2M checkpoint, and E2F pathways were significantly upregulated in clusters with several differentially enriched pathways.

**Discussion:**

This study elucidates the gene signatures associated with pyroptosis and immune infiltration in T2D and provides a critical resource for understanding of islet dysfunction and T2D pathogenesis.

## 1 Introduction

Diabetes mellitus is a chronic disease with high death and disability rates and large global economic burden. Type 2 diabetes (T2D) accounts for > 90% of all diabetes cases. The prevalence of T2D in China has increased rapidly in recent decades (1). A key pathological feature of T2D is hyperglycemia, which results from insulin resistance in peripheral tissues and islet beta cell dysfunction. Current therapies for T2D are directed toward reducing elevated blood glucose levels by improving insulin sensitivity in partial peripheral tissue, enhancing insulin secretion from the remaining beta cells, and promoting urinary glucose excretion. However, interventions focused on improving islet beta cell dysfunction are lacking. T2D is often associated with a strong genetic predisposition (2), but its genetics remain poorly understood (3). Understanding the diverse molecular processes and pathophysiological mechanisms, especially islet beta cell dysfunction, which triggers T2D is crucial to improve the prevention and treatment of this disease.

Islet dysfunction is an important pathophysiological mechanism in T2D (4). Islet inflammation plays a crucial role in islet dysfunction (5–7). This process is characterized by immune cell infiltration (7–9), cell death (10, 11), fibrosis (12), and amyloid deposition (13, 14). The link between islet inflammation and dysfunction has been well explored (15). Multiple studies on T2D have shown that targeting islet inflammation could help maintain normal islet function (9, 16). In addition, histological changes, including immune cell infiltration (8, 17) and pyroptosis (10), have been observed in the islets of patients with T2D.

Microarray is a promising and widely used method for large-scale gene expression profiling. Several studies have been performed to enhance our understanding of the molecular mechanisms underlying T2D pathogenesis. Recent studies have focused on the relationships between T2D-related genes and immune infiltration (18, 19) as well as between pyroptosis- and diabetes-related genes (20). Pyroptosis is a type of programmed cell death associated with inflammation and immunity (21). However, the roles of pyroptosis and infiltrating immune cells in islet dysfunction of patients with T2D have yet to be explored.

T2D is a chronic, heterogeneous, and progressive disease. Elucidating the molecular basis underlying islet dysfunction, which has been implicated in the pathogenesis of T2D, has been a major focus of diabetes research. Conventional bulk RNA sequencing (RNA-seq) measures the average RNA levels in samples. Advances in single-cell RNA sequencing (scRNA-seq) have enabled specific profiling of cell populations (22). Single-cell transcriptome analysis provides novel insights into cellular functional alterations that contribute to islet dysfunction and T2D pathogenesis (23) and may reveal cellular heterogeneity in T2D. In the present study, we aimed to identify novel biomarkers (genes related to disease phenotypes, pyroptosis, and immune infiltration) in T2D by performing a conjoint analysis of three bulk RNA-seq datasets of islets. We constructed miRNA and transcriptional networks based on the identified genes and performed functional analyses. We then analyzed the pyroptosis-related genes and infiltrating immune cells in different molecular subtypes of T2D. Furthermore, we analyzed single-cell data of islets to reveal the heterogeneity in T2D. This study elucidates the gene signatures associated with pyroptosis and immune infiltration in T2D and provides an important foundation for understanding of islet dysfunction and T2D pathogenesis.

## 2 Materials and methods

### 2.1 Data acquisition

Three bulk RNA-seq datasets (GSE118139, GSE25724, and GSE20966) were downloaded from the National Center for Biotechnology Information Gene Expression Omnibus (GEO) database. GSE118139 (24) was obtained using the GPL22120 platform Agilent-078298 human ceRNA array V1.0 4X180K [Probe Name Version] (*Homo sapiens*). GSE118139 contains data from four human islet samples, of which two were from patients with T2D and two were from patients without diabetes. GSE25724 (25) was obtained using the GPL96 platform [HG-U133A] Affymetrix Human Genome U133A Array. GSE25724 contains data from 13 human islet samples, of which six were from patients with T2D and seven were from patients without diabetes. GSE20966 (26) was obtained using the GPL1352 platform [U133_X3P] Affymetrix Human X3P Array. GSE20966 contains data from 20 human islet samples, of which 10 were from patients with T2D and 10 were from patients without diabetes. We downloaded the raw data from these three datasets and merged them into a matrix file containing 18 samples from patients with T2D and 19 samples from patients without diabetes. Batch effects were eliminated using the removeBatchEffect function of the limma package in R. The quality of the datasets was assessed using boxplots, principal component analysis (PCA), and heatmaps. After removing the batch effects, the merged matrix was used for subsequent analyses.

Additionally, we downloaded the single-cell transcriptomic dataset GSE153855 (27) from the GEO database. This dataset was obtained using the GPL16791 platform Illumina HiSeq 2500 (*Homo sapiens*). GSE153855 contains data from 11 human islet samples, of which five were from patients with T2D and six were from patients without diabetes.

### 2.2 Differential gene expression analysis

We performed a differential gene expression analysis of the merged dataset to compare the transcriptomes of the islet samples from patients with and without T2D. Data were analyzed using the limma package in R (version 3.52.2) (28). Differentially expressed genes (DEGs) related to T2D were defined as upregulated genes with a log fold change (FC) above 0.5 or downregulated genes with a logFC lower than -0.5 at *P* < 0.05.

### 2.3 Gene ontology (GO) and Kyoto Encyclopedia of Genes and Genomes (KEGG) enrichment analyses

GO (29) enrichment analysis including biological process, molecular function, and cellular component categories is a common method for large-scale functional enrichment studies of genes at different dimensions and levels. KEGG provides genomic and molecular information (30). KEGG pathway analysis is widely used in bioinformatics to annotate and enrich pathways. T2D-related DEGs were subjected to GO and KEGG pathway analyses using the clusterProfiler package in R (31), with *P* < 0.05 as a significance threshold. The results of the enrichment analyses were visualized using bubble plots.

### 2.4 Differential expression analysis according to ESTIMATE immune score

Estimation of STromal and Immune cells in MAlignant Tumor tissues using Expression Data (ESTIMATE) (32) is used to infer the proportion of stromal and immune cells *via* gene expression signatures. This algorithm was used to calculate the immune, stromal, and ESTIMATE scores. The samples were divided into high- and low-immune score groups according to their median immune scores, and the differential genes between the two groups were analyzed using the limma package in R (version 3.52.2) (28). Significant differential gene expression was defined as *P* < 0.05, and absolute values of logFC were > 0.05. Volcano and histogram plots were generated using the ggplot2 package in R (version 3.3.6) (33), and a heatmap was plotted using the pheatmap package in R (version 1.0.12) (34).

### 2.5 Expression analysis of pyroptosis-related genes

By querying the gene set of pyroptosis-related genes in the MSigDB (http://software.broadinstitute.org/gsea/msigdb) database (35, 36) and reviewing previous studies (37), we obtained 31 pyroptosis-related genes that were expressed in the merged matrix (**Table S1**). Heatmaps and boxplots were created to display the expression patterns of the 31 pyroptosis-related genes in the merged matrix. We then queried the chromosomal locations of these genes based on the human reference genome (UCSC.HG19.Human.CytoBandIdeogram) from the GENCODE database (38). The RCircos package in R (version 1.2.2) (39) was used to create a Circos plot for the expression distributions of the genes on the chromosome. A correlation-based heatmap was generated using the corrplot package in R (40), and correlation scatter plots were created using the ggpubr package in R (version 0.4.0) (41).

### 2.6 Construction of a clinical prediction model

The association between the genes and T2D was assessed through univariate logistic regression analysis and genes with *P* < 0.5 were selected for further least absolute shrinkage and selection operator (LASSO) regression. A nomogram was established using the results of LASSO regression to predict risk. A calibration curve was generated to evaluate the relationship between nomogram predictive probability and observed outcome. In addition, we constructed receiver operating characteristic (ROC) curves and calculated the areas under the ROC curve (AUCs) to assess the predictive performance of the model (R package pROC).

### 2.7 Gene set variation analysis (GSVA)

GSVA (42) is a nonparametric, unsupervised method for estimating variations in gene set enrichments through expression dataset samples. The pyroptosis pathway score in the merged matrix was measured using the GSVA package in R (version 1.42.0). The GSVA algorithm transforms gene expression data into a gene set sample matrix, producing an enrichment score for each sample and pathway. Each pathway gene set was computed using the Kolmogorov–Smirnov rank test statistic.

### 2.8 Gene set enrichment analysis (GSEA)

GSEA is used to identify classes of genes or proteins that are overrepresented in a large group of samples (43) and are highly correlated with disease phenotypes. The merged matrix was analyzed using GSEA to identify significantly enriched or depleted gene sets. Gene sets (msigdb.v7.0.entrez.gmt) were downloaded from the Molecular Signatures database (MSigDB) (16). GSEA was performed using the clusterProfiler package in R (version 4.4.4).

### 2.9 Weighted gene co-expression network analysis (WGCNA)

WGCNA (44) is a widely used data mining method to construct biologically relevant modules based on pairwise correlations between gene expression profiles. Genes with the top 25% variance of gene expression values were screened for cluster analysis following the WGCNA tutorial (https://horvath.genetics.ucla.edu/html/CoexpressionNetwork/Rpackages/WGCNA/Tutorials/). An appropriate soft threshold was selected to calculate the adjacency matrix, which was converted to a topological overlap matrix (TOM). Then, we hierarchically clustered this TOM and used the cutreeDynamic function with method ‘tree’ to identify modules of correlated genes (minimum module size of 30 genes). The pathways enriched by GSVA were fused with pyroptosis-related modules to observe the correlation between each module and pyroptosis. A correlation heatmap was drawn to obtain the gene sets corresponding to the modules positively correlated with pyroptosis.

### 2.10 Protein–protein interaction (PPI) establishment and identification of hub genes

Considering the important roles of pyroptosis and immunity in diabetes, we analyzed whether any genes of the pyroptosis module overlapped with T2D-related DEGs between the high- and low-immune score groups. A PPI network based on overlapping genes was constructed using the STRING database (45). PPI pairs were identified using the confidence criterion (0.75). The degree of each node was calculated using the CytoHubba plugin for Cytoscape (version 3.7.1) (46). A bar chart was drawn according to the reverse order of the degree of each node. Genes with more than 10 nodes were selected as hub genes.

### 2.11 Construction of miRNA interaction network

Candidate target miRNAs were predicted using the miRTarBase database and analyzed using the multiMiR package in R (version 1.18.0) (47). Photoactivatable ribonucleoside crosslinking and immunoprecipitation (PAR-CLIP) (48) is used to identify the binding sites of RNA-binding proteins and miRNA-containing ribonucleoprotein complexes. The miRNA validation level was set to “PAR-CLIP” to further screen miRNAs that interact with hub genes. Finally, a miRNA–mRNA interaction network based on the above prediction results was constructed using Cytoscape (version 3.7.1) (46).

### 2.12 Construction of transcriptional regulatory network

Transcription factor (TF) lists were retrieved from the Cistrome database (49, 50)(http://cistrome.org/), and differential gene expression analysis of the merged dataset was performed to identify differentially expressed TFs. A correlation test was performed to identify TFs associated with hub genes, with an absolute value of correlation coefficient > 0.4 and *P* < 0.001. The resulting data were imported into Cytoscape (version 3.7.1) to construct a transcriptional regulatory network.

### 2.13 Analysis of immune subtypes

The immune subtypes of each sample were predicted from the merged gene expression profiles using the ImmuneSubtypeClassifier package in R (version 0.1.0). A Sankey diagram was generated to show the relationship between hub genes and six immune subtypes using the ggalluvial package in R (version 0.12.3). The six immune subtypes were wound healing (C1), IFN-γ dominant (C2), inflammatory (C3), lymphocyte depleted (C4), immunologically quiet (C5), and transforming growth factor (TGF)-β dominant (C6).

### 2.14 Analysis of immune cell infiltration

To determine the relative abundance of 22 immune cells in the merged matrix, we analyzed the transcriptomic data using CIBERSORT (51). For each sample, the sum of all the estimated fractions of immune cells was equal to one. Differences in immune cell abundances between the high-risk and low-risk groups were compared using the t-test, and *P* < 0.05 was considered to indicate statistical significance. A correlation-based heatmap was generated using the corrplot package in R. Correlations were calculated using the Pearson’s correlation coefficient. Scatter plots and fitting curves were constructed using the ggplot2 package in R. The merged matrix was subjected to single-sample gene set enrichment analysis (ssGSEA) using the GSVA package in R (version 1.42.0). The infiltration levels of 28 subpopulations of tumor-infiltrating lymphocytes were evaluated based on the cell marker gene CellMarker (52). Box plots were constructed using pubr.

### 2.15 Unsupervised clustering of T2D samples

Owing to the prevalence of heterogeneity between patients, unsupervised clustering of T2D samples based on 17 hub genes could resolve this heterogeneity and reclassify the samples. Unsupervised consensus clustering of the samples was performed through aggregation hierarchical clustering using the ConsensusClusterPlus package in R (version 1.60.0). Spearman’s method was used to calculate the distance, and clustering was conducted using K-means. We ascertained the optimal number of clusters by considering a consensus matrix heatmap, consensus cumulative distribution functions (CDFs), and the relative change in area under the CDF curve. Boxplots and heatmaps were drawn to determine the expression differences of hub genes, immune scores, and immune cell infiltration levels among different clusters.

### 2.16 Quality control, cluster analysis, and major cell type identification of single-cell expression data

The single-cell RNA sequencing dataset GSE153855 was imported into R and converted into a Seurat object using the Seurat package in R (version 4.1.1) (53). A high proportion of transcript counts derived from mitochondria-encoded genes might indicate low cell quality; therefore, we removed cells with a percentage of mitochondrial transcripts larger than 5%. We conducted quality control through the counts and expression of sequencing genes and the percentage of mitochondrial genes. Cells were filtered using nFeature_RNA > 200, nCount_RNA < 6000, and percent.mt < 5 as cutoffs. Violin plots were created to show the number of genes, gene expression values, and percentage of mitochondrial genes. Dimensionality was reduced using PCA. The first 10 principal components were chosen to further reduce dimensionality and visualization using the t-distributed statistical neighbor embedding (t-SNE) algorithm. The cell type information for each cluster was annotated using built-in annotations from the GSE153855 dataset. The reliability of the built-in annotation information was visualized by creating bubble and violin plots, which displayed the expression of marker genes reported in the literature for various cell types of islets (54–57) in the clusters. We used the following markers for cell type identification: beta cells (*FXYD2*), alpha cells (*KANSL3* and *SOD2*), delta cells (*LAPTM4B*, *TMEM163*, and *UBR4*), macrophages (*CD86*), endothelial cells (*FLT1*), and ducts (*PROM1*).

### 2.17 Differential analysis of hub genes among different cell types

DEGs were identified among all clusters using the “FindAllMarkers” function, which uses the Wilcoxon rank-sum test. The hub genes among the clusters were screened by taking the intersection DEGs between the clusters and cluster marker genes. The DoHeatmap function was used to generate an expression heatmap for hub gene expression.

### 2.18 Pseudotime trajectory analysis

The differentiation pseudotime of the different cell subtypes was inferred using the Monocle package in R (version.2.22.0) (58). Highly variable genes were identified using the “VariableFeatures” function, and cells were ranked using the “setOrderingFilter” function. Finally, the “DDRTree” method was used to reduce dimensionality, and the “orderCells” function was used to estimate the arrangement of cells along the trajectory. Plots of cellular trajectories were drawn based on marker genes and clusters. Each trajectory was analyzed using a standard protocol with default parameters.

### 2.19 Cell–cell communication

The CellChat package in R (54) was used to infer and qualify intercellular communication by combining single-cell expression profiles with known ligands, receptors, and their cofactors. The ligand–receptor interaction probability and perturbation test were used to identify significant ligand–receptor relationship pairs. Cell–cell communication networks were then integrated by adding the number or strength of ligand–receptor pairs with significant interactions between cell types. A heatmap was used to show the contribution of the input and output pathways to the cells. The numbers and weights of the interactions are shown by circular plots.

### 2.20 Statistical analyses

All data calculations and statistical analyses were performed using R. For the comparison of two groups of continuous variables, the statistical significance of normally distributed variables was estimated using the independent t-test, and differences between non-normally distributed independent variables were analyzed using the Wilcoxon rank-sum test. Chi-square test or Fisher’s exact test was used to compare and analyze the statistical significance between two groups of categorical variables. The correlation coefficients between different genes were calculated using Pearson correlation analysis. All statistical tests were 2-sided, and *P* < 0.05.

**Figure 1:**
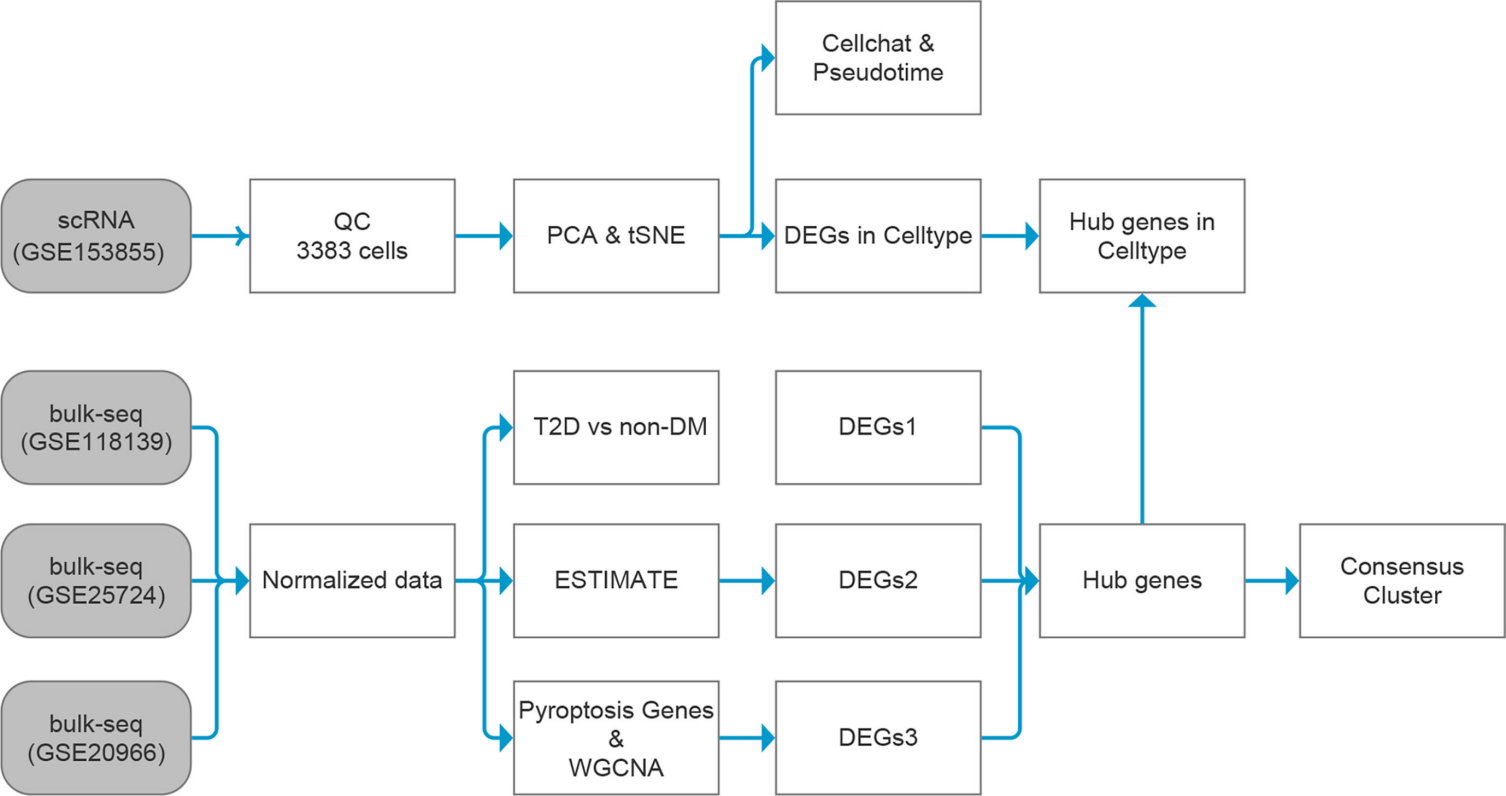
Data analysis flow chart. T2D, type 2 diabetes; non-DM, non-diabetes mellitus, ESTIMATE, Estimation of STromal and Immune cells in MAlignant Tumor tissues using Expression data; scRNA, single-cell RNA; WGCNA, weighted gene co-expression network analysis; DEGs, differentially expressed genes.

The general idea and methodologies used in this study are shown in a flow chart (**Figure 1**).

## 3 Results

### 3.1 Functional analysis of T2D-related genes and ESTIMATE immune score

**Figure 2:**
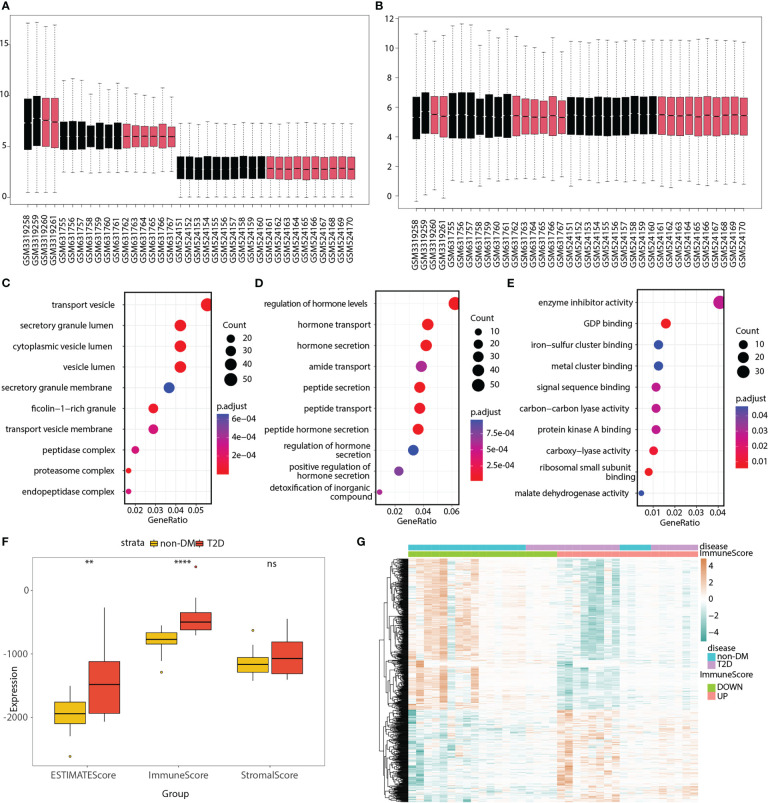
GO analyses of T2D-related genes; ESTIMATE immune score, and 550 immune and T2D-related genes in the merged matrix. **(A)** Box plot of three datasets without batch effect correction. **(B)** Box plot of three datasets after batch effect correction. Red represents T2D patient, whereas black represents non-DM control. **(C–E)** Top 10 significantly enriched cellular components, biological processes, and molecular functions. **(F)** Box plot showing the differences in ESTIMATE, immune, and stromal scores between the T2D and non-DM groups. **(G)** Heatmap showing the expression differences of 550 immune and T2D-related genes between the low- and high-immune score groups as well as between the T2D and non-DM groups. ns, P ≥ 0.05; *, P < 0.05; **, P < 0.01; ***, P < 0.001; ****, P < 0.0001.

Three bulk RNA-seq datasets (GSE118139, GSE25724, and GSE20966) were combined into a merged matrix, with 18 islet samples from patients with T2D and 19 islet samples from patients without diabetes. Heatmaps (**Figures S1A, B**), box plots (**Figures 2A, B**), and PCA plots (**Figures S2A, B**) indicated the successful removal of batch effects from the merged matrix, which was then used for subsequent analyses. A total of 918 T2D-related DEGs (220 upregulated and 698 downregulated) were identified. GO terms were analyzed, as shown in **Figures 2C–E** and **Table S2**, to explore the functions of the DEGs.

The box plot shows the differences in the ESTIMATE, immune, and stromal scores between the T2D and non-diabetes mellitus (non-DM) groups (**Figure 2F**). The immune score was significantly higher in the T2D group than in the non-DM group. Based on the median value of the immune score, the samples were divided into two groups (high- and low-immune score groups). The volcano plot (**Figure S2C**) shows that 835 immune-related genes were differentially expressed between the high- and low-immune score groups. The Venn diagram displays 550 immune and T2D-related genes (**Figure S2D**; **Table S3**). The heatmap (**Figure 2G**) shows the expression differences of these genes between the low- and high-immune score groups as well as between the T2D and non-DM groups.

### 3.2 Panorama of pyroptosis-related genes and correlation analysis of pyroptosis-related genes in T2D

**Figure 3:**
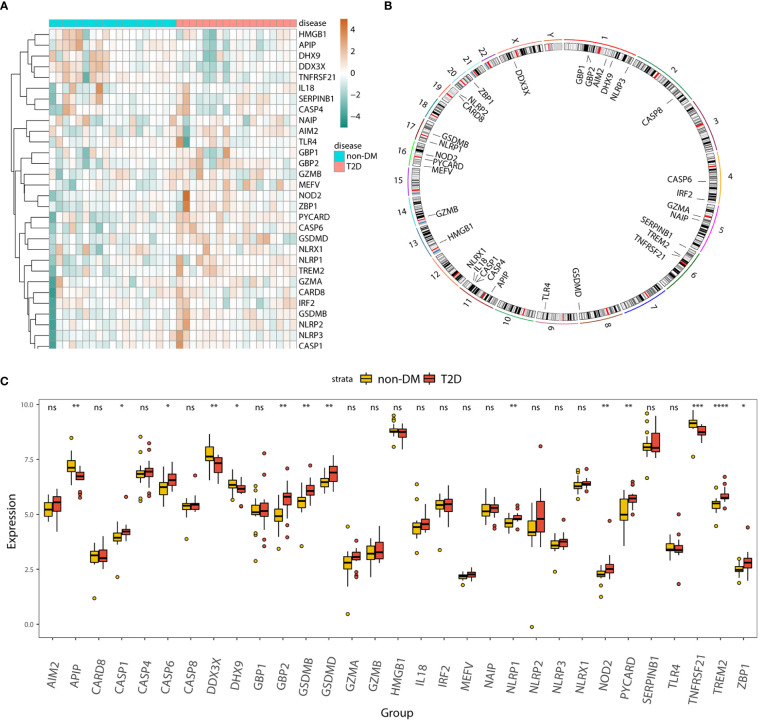
Panorama of pyroptosis-related genes in T2D. **(A)** Heatmap displaying the expression patterns of 31 pyroptosis-related genes in the T2D and non-DM groups. **(B)** Circos plot showing the distribution of 31 pyroptosis-genes on the chromosome. **(C)** Box plot displaying the expression patterns of 31 pyroptosis-related genes in the T2D and non-DM groups. ns, P ≥ 0.05; *, P < 0.05; **, P < 0.01; ***, P < 0.001; **** , P < 0.0001.

The heatmap (**Figure 3A**) and boxplot (**Figure 3C**) display the expression patterns of the 31 pyroptosis-related genes in the T2D and non-DM groups. *APIP*, *DDX3X*, *DHX9*, and *TNFRSF21* were significantly downregulated in the T2D group, whereas *CASP1*, *GBP2*, *GSDMB*, *GSDMD*, *NLRP1*, *NOD2*, *PYCARD*, *TREM2*, and *ZBP1* were significantly upregulated in the T2D group. The Circos plot (**Figure 3B**) shows the expression distributions of the 31 pyroptosis-related genes on the chromosome.

We analyzed the association between the 31 pyroptosis-related genes and T2D. The correlation heatmap (**Figure S3A**) shows the correlated expression patterns of the 31 pyroptosis-related genes. The top four significant negative and positive correlations of gene pairs are shown by correlation scatter plots (**Figures S3B–I**): *AIM2-HMGB1* (r = -0.628, *P* < 0.001; **Figure S3B**), *DDX3X*-*TREM2* (r = -0.614, *P* < 0.001; **Figure S3C**), *HMGB1-TREM2* ((r = -0.611, *P* < 0.001; **Figure S3F**), *NLRX1-DHX9* (r = -0.719, *P* < 0.001; **Figure S3I**), *CASP1-CASP8* (r = 0.844, *P* < 0.001; **Figure S3D**), *NOD2-ZBP1* (r = 0.843, *P* < 0.001; **Figure S3E**), *NLRP3-CASP1* (r = 0.794, *P* < 0.001; **Figure S3G**), and *GSDMB*-*NLRP2* (r = 0.78, *P* < 0.001; **Figure S3H**).

### 3.3 Construction of the clinical prediction model

**Figure 4:**
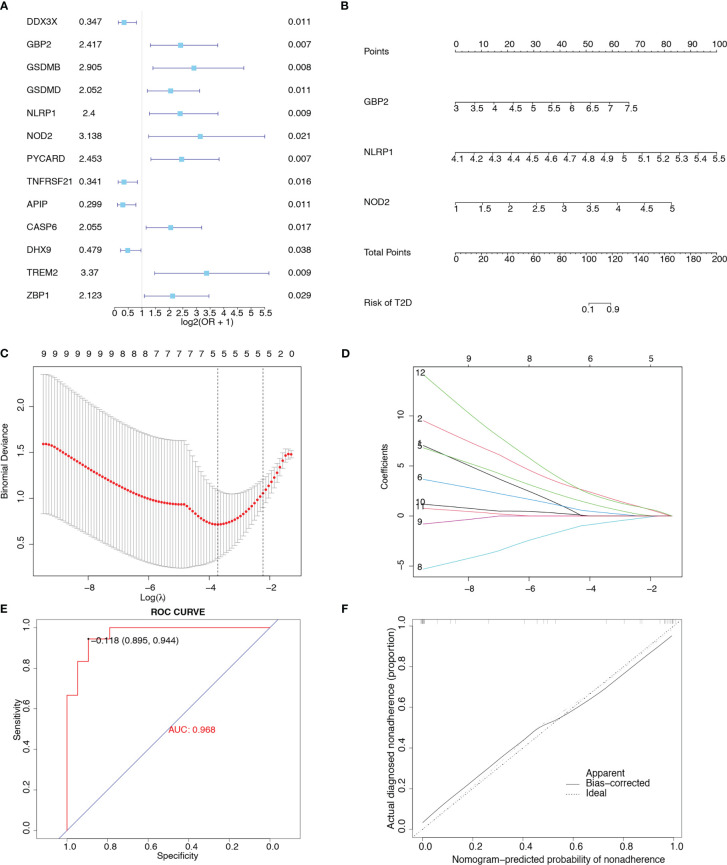
Construction of a clinical prediction model. **(A)** Univariate forest plot showing the predictive power of 31 pyroptosis-related genes for T2D. **(B)** Nomogram integrating *GBP2*, *NLRP1*, and *NOD2* in T2D. **(C)** Diagram representing relationships between penalty parameters and binominal deviances. **(D)** Diagram representing the relationships between penalty parameters and regression coefficients. **(E)** ROC curve of the nomogram (AUC = 0.968). **(F)** Calibration curve of the nomogram.

We evaluated the predictive power of the 31 pyroptosis-related genes for T2D. As shown in the univariate forest plot (**Figure 4A**), 13 pyroptosis-related genes were significantly associated with the prevalence of T2D: *DDX3X*, *GBP2*, *GSDMB*, *GSDMD*, *NLRP1*, *NOD2*, *PYCARD*, *TNFRSF21*, *APIP*, *CASP6*, *DHX9*, *TREM2*, and *ZBP1*. LASSO regression was performed on these 13 genes. The lambda value with the minimal average deviance was determined as the optimal lambda (-3.7) through cross-validation (**Figures 4C, D**). *GBP2*, *NLRP1*, and *NOD2* were identified, and a nomogram (**Figure 4B**) was constructed to predict the probability of T2D. ROC analysis revealed that this risk score for T2D had high predictive power, with an AUC of 0.968 (**Figure 4E**). The calibration curve (**Figure 4F**) also demonstrated the accuracy of the nomogram-predicted probability.

### 3.4 WGCNA

**Figure 5:**
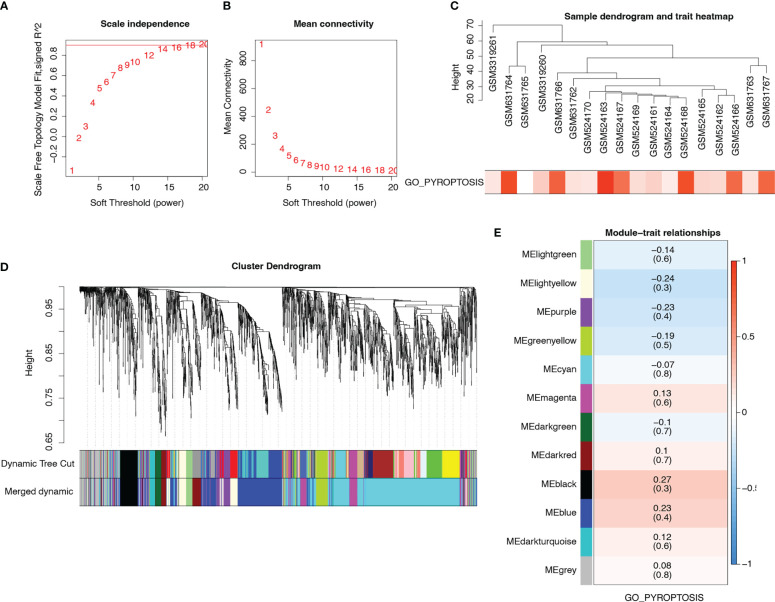
WGCNA. **(A)** Analysis of scale-free index for various soft-threshold powers. **(B)** Analysis of mean connectivity for various soft-threshold powers. **(C)** Dendrogram of samples and heatmap of pyroptosis trait. **(D)** Cluster dendrogram plots of the 12 coexpressed modules identified by WGCNA in different colors. **(E)** Heat map describing the relevance of modules (rows) to pyroptosis (columns). Blue represents negative correlations, whereas red represents positive correlations.

Data from patients with T2D in the merged matrix were used as inputs for WGCNA. Meanwhile, we performed GSVA and extracted the pyroptosis-related pathway GO_PYROPTOSIS into WGCNA. We selected β = 14 as the soft thresholding power to ensure a scale-free network (**Figures 5A, B**). **Figure 5C** shows the trait and GO_PYROPTOSIS pathway scores for each sample. We constructed a hierarchical clustering tree and identified 12 modules after fusing them (**Figure 5D**). Among the 12 modules, six (magenta, darked, black, blue, dark turquoise, and grey) had 1193 genes that were positively correlated with the GO_PYROPTOSIS pathway (**Figure 5E**).

### 3.5 PPI network establishment and identification of pyroptosis-related hub genes

**Figure 6:**
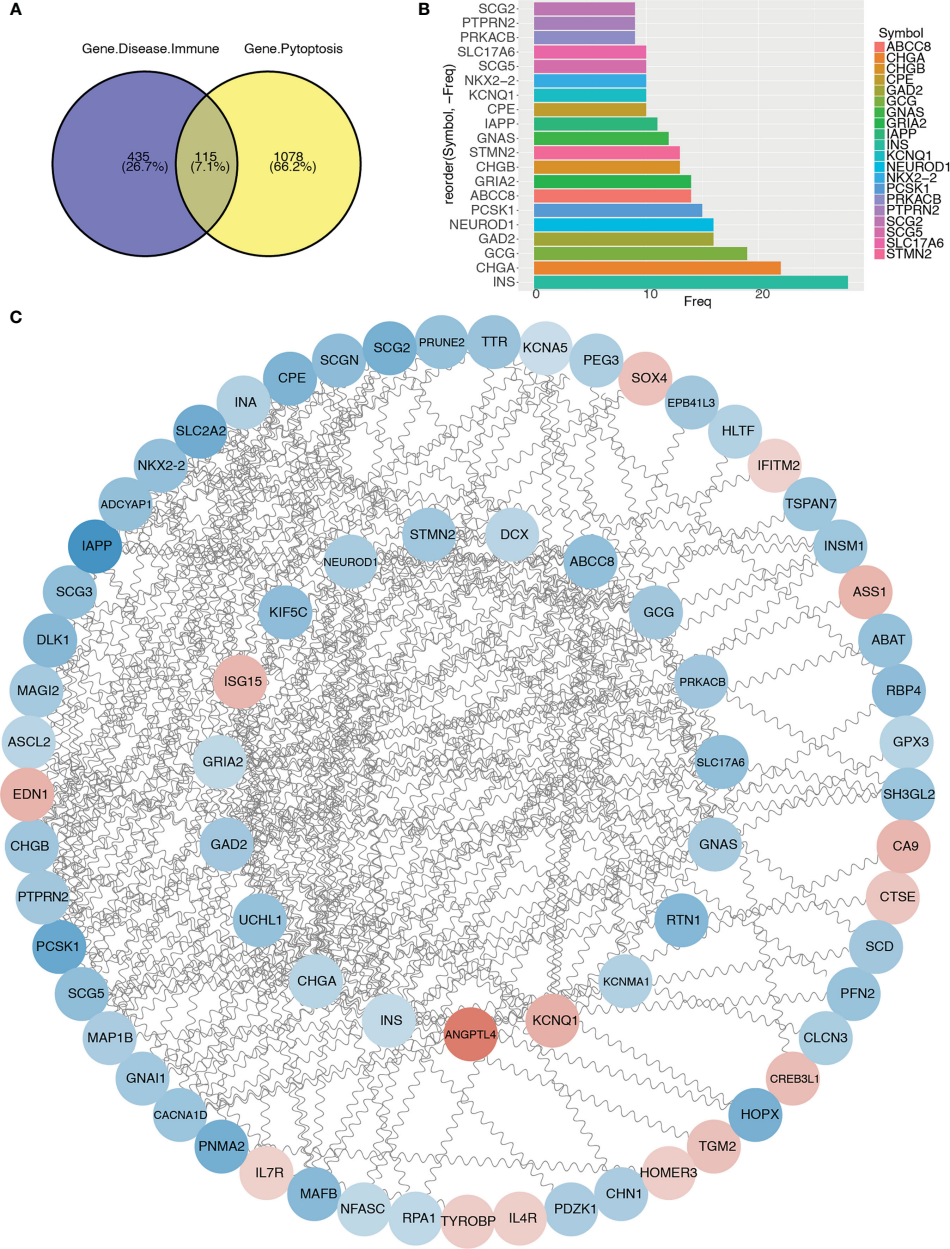
Protein–protein interaction network and identification of pyroptosis-related hub genes. **(A)** Venn diagram showing the intersection between immune and T2D-related genes with genes in pyroptosis-related modules. **(B)** Bar graph showing the top 20 genes in the intersection. **(C)** Protein–protein interaction network of genes in the intersection.

To further analyze the differentially expressed immune and T2D-related genes in the pyroptosis-related modules, we intersected 1193 genes in the pyroptosis-related modules with 550 immune and T2D-related DEGs and obtained 115 genes (**Figure 6A**; **Table S4**). Then, we constructed a PPI network using the STRING database and visualized the network using Cytoscape (**Figure 6C**). We identified the top 20 genes based on the number of nodes (**Figure 6B**) and then selected the top 17 genes with > 10 nodes as pyroptosis-related hub genes: *INS*, *CHGA*, *GCG*, *GAD2*, *NEUROD1*, *PCSK1*, *ABCC8*, *GRIA2*, *CHGB*, *STMN2*, *GNAS*, *IAPP*, *CPE*, *KCNQ1*, *NKX2-2*, *SCG5*, and *SLC17A6*.

### 3.6 Construction of miRNA interaction and transcriptional regulatory network

**Figure 7:**
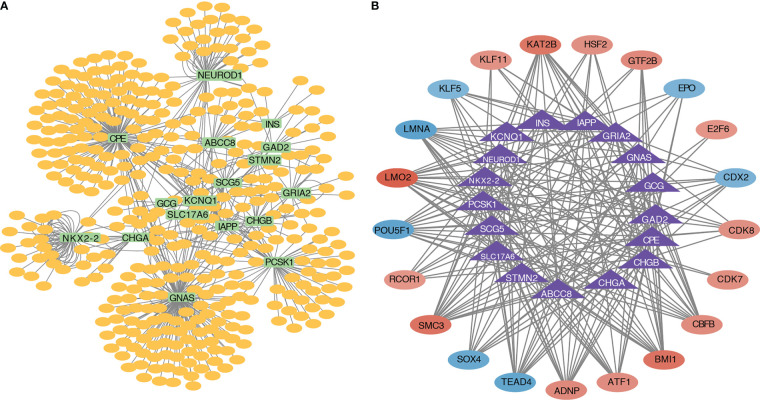
miRNA–mRNA interaction network and transcriptional regulatory network. **(A)** Interaction network diagram of pyroptosis-related hub genes and miRNAs. **(B)** Interaction network diagram of pyroptosis-related hub genes and transcription factors.

A miRNA–mRNA interaction network based on the 17 pyroptosis-related hub genes was constructed, and 635 miRNAs were identified. The miRNA–mRNA interaction network is shown in **Figure 7A**. A transcriptional regulatory network (**Figure 7B**) based on the 17 pyroptosis-related hub genes was constructed. The transcriptional regulatory network is shown in **Figure 7B**.

### 3.7 Enrichment analyses of pyroptosis-related hub genes

**Figure 8:**
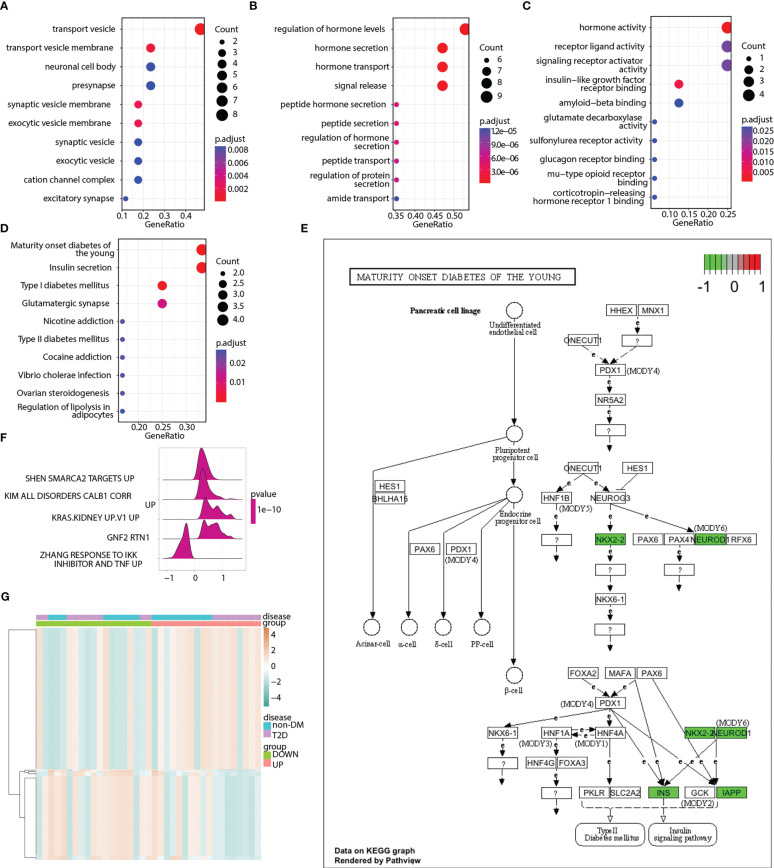
Enrichment analyses of pyroptosis-related hub genes. **(A)** Enriched GO terms in the “cellular component” category. **(B)** Enriched GO terms in the “biological process” category. **(C)** Enriched GO terms in the “molecular function” category. **(D)** Top 10 significantly enriched KEGG pathways of pyroptosis-related hub genes. **(E)** Abnormal expression of pyroptosis-related hub genes in maturity-onset diabetes of the young signaling pathway. **(F)** Enrichment map showing the top 5 pathways from pyroptosis score-based GSEA. X-axis represents enrichment score, and Y-axis represents pathway name. **(G)** Heatmap illustrating the enriched pathways between the low- and high-pyroptosis score groups as well as between the T2D and non-DM groups. Red represents upregulation, whereas green represents downregulation.

GO terms were analyzed to explore the functions of pyroptosis-related hub genes (**Figures 8A–C**). KEGG pathway analysis indicated that the pyroptosis-related hub genes were enriched in maturity-onset diabetes of the young, insulin secretion, type 1 diabetes mellitus, and glutamatergic synapses (**Figure 8D**). The signaling pathway of maturity-onset diabetes of the young is shown in **Figure 8E**. Four hub genes (*NKX2-2*, *NEUROD1*, *INS*, and *IAPP*) were significantly downregulated. The ssGSEA algorithm was used to calculate the pyroptosis score of each sample in the merged matrix based on the 17 pyroptosis-related hub genes, and the samples were divided into high- and low-pyroptosis score groups according to the median pyroptosis score. GSEA (**Table S5**) and GSVA (**Table S6**) were performed to compare the high- and low-pyroptosis score groups. The top five pathways in the GSEA results are shown in **Figure 8F**. The heatmap of the GSVA pathway enrichment results shows the resulting spectrum of the differential enrichment of 177 pathways in the high- and low-pyroptosis score groups (**Figure 8G**).

### 3.8 Analyses of immune subtypes and correlation analyses of infiltrating immune cells

**Figure 9:**
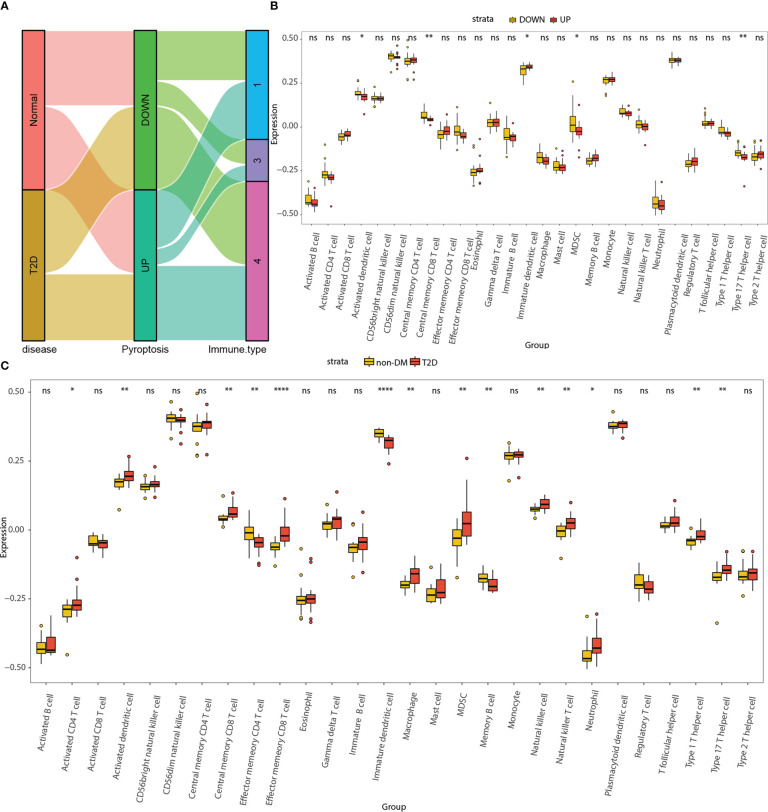
Correlation analyses among pyroptosis-related hub genes, immune subtypes, and infiltrating immune cells. **(A)** Sankey diagram demonstrating the association among disease states, pyroptosis states, and immune subtypes. **(B)** Differential distributions of 28 types of infiltrating immune cells between the high- and low-pyroptosis score groups. **(C)** Differential distributions of 28 types of infiltrating immune cells between the non-DM and T2D groups. Red represents upregulation, while yellow represents downregulation.ns, *P* ≥ 0.05; *, *P* < 0.05; **, *P* < 0.01; ***, *P* < 0.001; **** , P < 0.0001.

The Sankey diagram demonstrates the association between disease states, pyroptosis states, and immune subtypes (**Figure 9A**). The disease states were almost uniformly distributed among pyroptosis states and the C1, C3, and C4 immune subtypes.

The differential distributions of 28 types of infiltrating immune cells were compared between the high- and low- pyroptosis score groups as well as between the T2D and non-DM groups. In the high pyroptosis score group (UP), the abundance of activated dendritic cells, central memory CD4+ T cells, myeloid-derived stem cells (MDSCs), and type 17 T helper cells significantly decreased, whereas the abundance of immature dendritic cells significantly increased (**Figure 9B**). In the T2D group, the abundance of activated CD4+ T cells, activated dendritic cells, central memory CD8+ T cells, effector memory CD8+ T cells, macrophages, MDSC, natural killer cells, natural killer T cells, neutrophils, type 1 T helper cells, and type 17 T helper cells significantly increased, whereas the abundance of effector memory CD4+ T cells, immature dendritic cells, and memory B cells significantly decreased (**Figure 9C**).

We evaluated the infiltration of 22 immune cell types in the merged matrix by using the CIBERSORT algorithm and determined the abundance of 13 immune cell types. The correlation heatmap depicted possible correlations between the 13 types of immune cells (**Figure S4A**). The significant negative and positive correlations between the two types of immune cells are shown in **Figures S4B–F**.

### 3.9 Analysis of pyroptosis-related hub genes and immune infiltration in different molecular subtypes

We classified the T2D samples into a merged matrix through unsupervised consensus clustering. The optimal number of clusters was determined to be two after comprehensive consideration of the delta area curve, CDF, and consensus matrix heatmap (**Figures S5A–C**).

**Figure 10:**
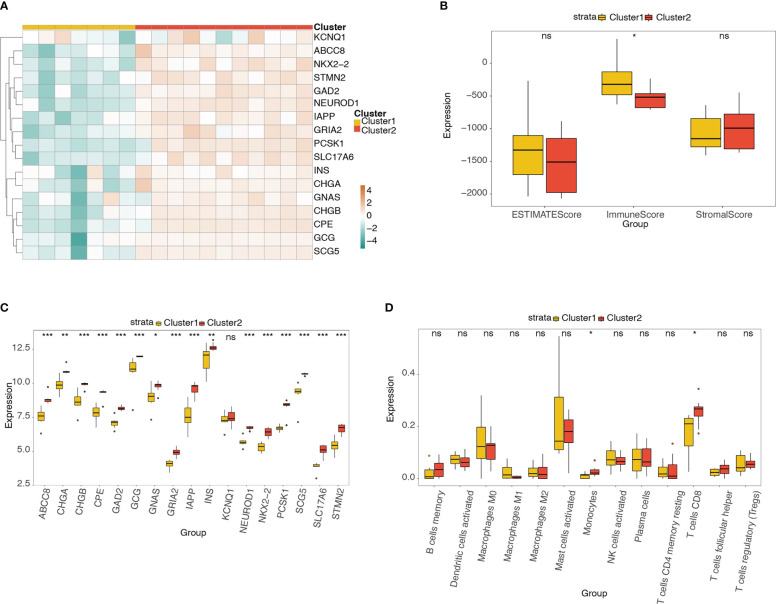
Analysis of pyroptosis-related hub genes and infiltrating immune cells in different molecular subtypes. **(A)** Heatmap of the expression distribution of pyroptosis-related hub genes in different molecular subtypes (clusters 1 and 2). **(B)** Box plot showing the differences in ESTIMATE, immune, and stromal scores between clusters 1 and 2. **(C)** Box plot of the expression distribution of pyroptosis-related hub genes between clusters 1 and 2. **(D)** Box plot showing the difference in 13 types of immune cells between clusters 1 and 2. ns, *P* ≥ 0.05; *, *P* < 0.05; **, *P* < 0.01; ***, *P* < 0.001.

The heatmap (**Figure 10A**) and box plot (**Figure 10C**) reveal the expression distributions of the pyroptosis-related hub genes in different molecular subtypes (clusters 1 and 2). The expression of 17 pyroptosis-related hub genes was high in cluster 2 and low in cluster 1. The box plot shows the difference in ESTIMATE, immune, and stromal scores between clusters 1 and 2 (**Figure 10B**). The immune score was significantly higher in cluster 1 than in cluster 2. **Figure 10D** shows the differences in the abundance of the 13 types of immune cells between the two clusters. The abundance of monocytes and CD8+ T cells was significantly higher in cluster 2 than in cluster 1.

### 3.10 Correlation analysis between pyroptosis-related hub genes and infiltrating immune cells

**Figure 11:**
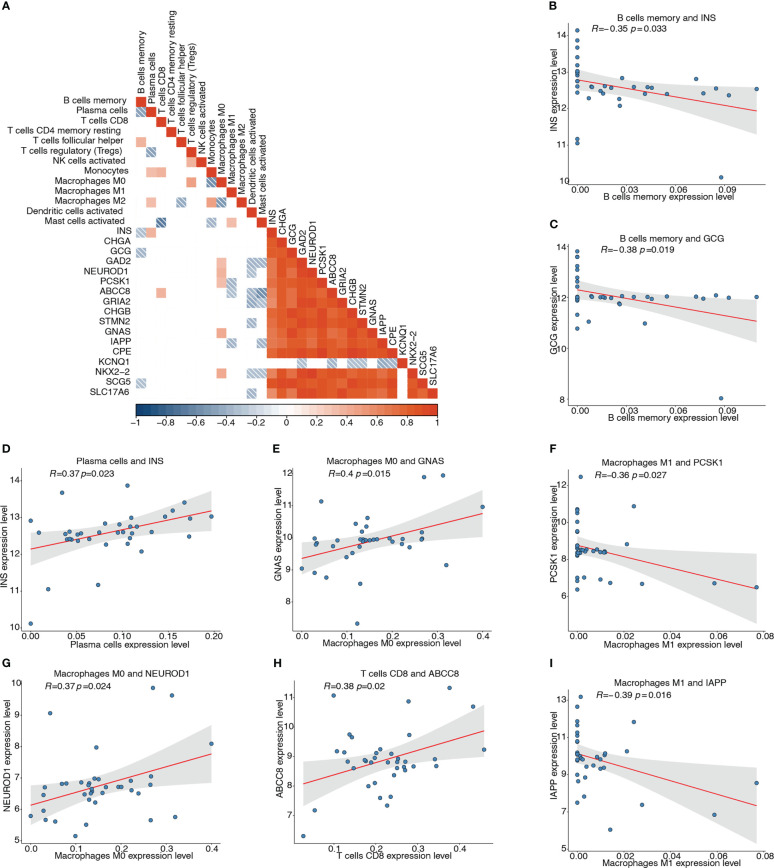
Correlation analyses between pyroptosis-related hub genes and infiltrating immune cells. **(A)** Correlation heatmap depicting possible correlations between 17 hub genes and the 13 types of immune cells. Red represents positive correlation, blue represents negative correlation, and white represents no significant correlation. **(B)** Correlation of memory B cells and INS (r = -0.351, *P* < 0.05). **(C)** Correlation of memory B cells and GCG (r = -0.385, *P* < 0.05). **(D)** Correlation of plasma cells and INS (r = 0.374, *P* < 0.05). **(E)** Correlation of M0 macrophages and GNAS (r = 0.397, *P* < 0.05). **(F)** Correlation of M1 macrophages and PCSK1 (r = -0.363, *P* < 0.05). **(G)** Correlation of M0 macrophages and NEUROD1 (r = 0.371, *P* < 0.05). **(H)** Correlation of CD8+ T cells and ABCC8 (r = 0.38, *P* < 0.05). **(I)** Correlation of M1 macrophages and IAPP (r = -0.395, *P* < 0.05).

We then analyzed the association of the 17 pyroptosis-related hub genes with the 13 types of infiltrating immune cells. The correlation heatmap depicts the possible correlations between the 17 hub genes and 13 types of immune cells (**Figure 11A**). The top four significant negative and positive correlations are shown by correlation scatter plots (**Figures 11B–I**): B cell memory and *INS* (r = -0.351, *P* < 0.05; **Figure 11B**), B cell memory and *GCG* (r = -0.385, *P* < 0.05; **Figure 11C**), macrophages M1 and *PCSK1* (r = -0.363, *P* < 0.05; **Figure 11F**), macrophages M1 and *IAPP* (r = -0.395, *P* < 0.05; **Figure 11I**), plasma cells and *INS* (r = 0.374, *P* < 0.05; **Figure 11D**), macrophages M0 and *GNAS* (r = 0.397, *P* < 0.05; **Figure 11E**), macrophages M0 and *NEUROD1* (r = 0.371, *P* < 0.05; **Figure 11G**), and T cells CD8 and *ABCC8* (r = 0.38, *P*< 0.05; **Figure 11H**).

### 3.11 Quality control, cluster analysis, and major cell-type identification of single-cell expression data

**Figure 12:**
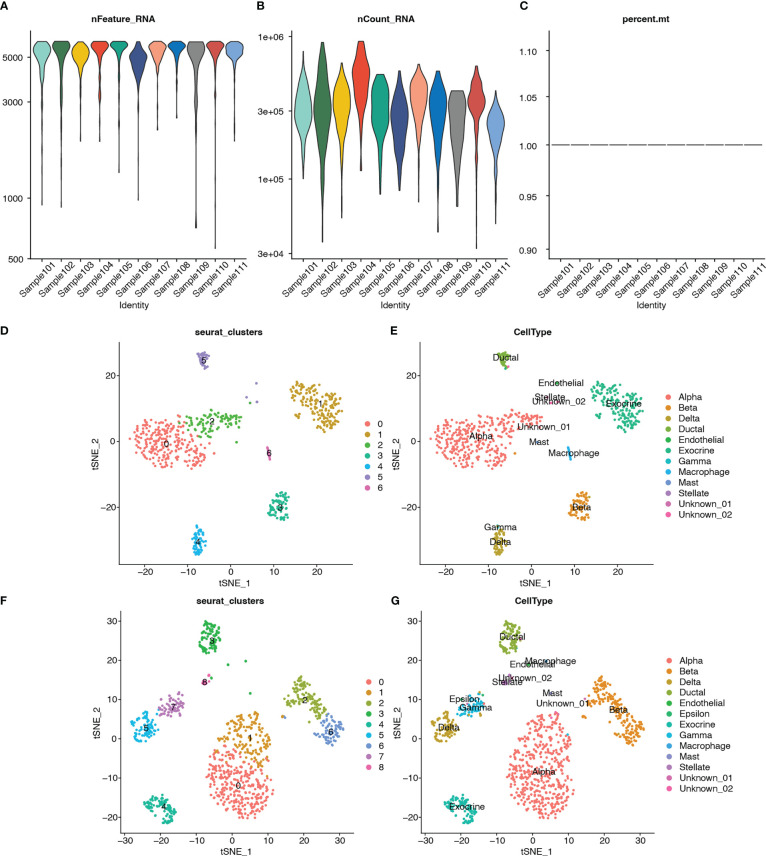
Quality control, cluster analysis, and major cell type identification of single-cell expression data GSE153855. **(A)** Violin plot showing numbers of genes (nFeature) of samples. **(B)** Violin plot showing expression values of genes (nCount) of samples. **(C)** Violin plot showing percent of mitochondria genes (percent.mt) of samples. **(D)** Dimensionality reduction plot using t-SNE showing seven distinct clusters of T2Dsamples. **(E)** Dimensionality reduction plot using t-SNE showing annotated the cell-type for each cluster of T2D samples. **(F)** Dimensionality reduction plot using t-SNE showing nine distinct clusters of non-T2D samples. **(G)** Dimensionality reduction plot using t-SNE showing annotated the cell type for each cluster of non-T2D samples.

Single-cell RNA-seq analysis was performed on five T2D and six non-T2D samples from the GSE153855 dataset. Cells were filtered using nFeature_RNA >200, nCount_RNA < 6000, and percent.mt < 5 as cutoffs. The violin plots show the number of genes (nFeature), expression values of genes (nCount), and percentage of mitochondrial genes (percent.mt) (**Figures 12A–C**). Subsequently, we performed PCA for dimensionality reduction to visualize the overall distribution of the data (**Figure S6A**) and the relationship between the number of principal components and standard deviation (**Figure S6B**). In this study, we selected 10 principal components for cell clustering. Eleven distinct clusters were identified among 1892 cells using t-SNE (**Figure S6C**). **Figure S6D** shows the clusters for the T2D and non-T2D samples.

**Figure 13:**
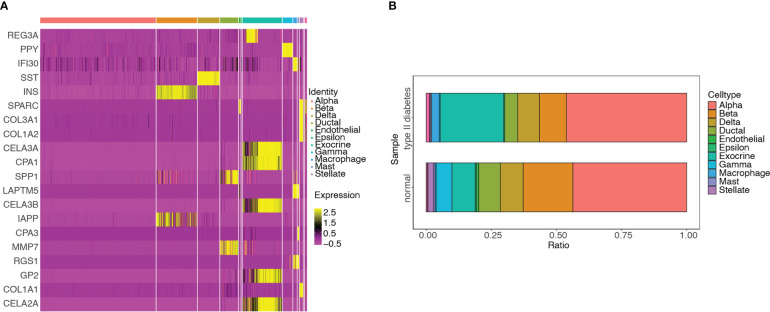
Differential analyses of hub genes among different cell types and distribution of the various cell types in T2D and non-T2D samples. **(A)** Heatmap of hub genes among 11 clusters. **(B)** Distribution of various cell types in T2D and non-T2D samples.

In addition, clustering analysis was performed separately using t-SNE for the T2D and non-T2D samples. The cell types for each cluster were annotated using built-in annotations in the GSE153855 dataset. The bubble and violin plots show the reliability of the built-in annotation information (**Figures S2E–F**). The respective marker genes were relatively highly expressed in the corresponding cells, which proved that the built-in cell annotations in this dataset were reliable. Seven distinct clusters were identified among 760 cells from the T2D samples through t-SNE (**Figure 12D**). **Figure 13E** shows the visualization of t-SNE colored according to the cell type in the T2D samples. As shown in **Figures 12D, E**, the cell types distributed in the seven clusters were as follows: alpha in cluster 0 (252, 98.824%), exocrine in cluster 1 (183, 100%), alpha in cluster 2 (99, 94.286%), beta in cluster 3 (76, 98.701%), delta in cluster 4 (62, 95.385%), ductal in cluster 5 (38, 71.698%), and macrophage in cluster 6 (22, 100%). Meanwhile, nine distinct clusters were identified among 1132 cells from the non-T2D samples through t-SNE (**Figure 12F**). **Figure 12G** shows the visualization of t-SNE colored according to the cell type in the non-T2D samples. As shown in **Figures 12F, G**, the cell types distributed in the nine clusters were as follows: alpha in cluster 0 (330, 100%), alpha in cluster 1 (164, 95.906%), beta in cluster 2 (126, 99.213%), ductal in cluster 3 (94, 78.333%), exocrine in cluster 4 (100, 100%), delta in cluster 5 (92, 97.872%), beta in cluster 6 (87, 98.864%), gamma in cluster 7 (66, 83.544%), and stellate in cluster 8 (23, 100%).

### 3.12 Differential analysis of hub genes among different cell types

DEGs were screened among the 11 cell types. The hub genes in the different cell types were identified by intersecting DEGs with 115 genes and visualized using a heatmap (**Figure 13A**). The distributions of various cell types in the T2D and non-T2D samples were also visualized (**Figure 13B**). The cell types distributed in the T2D samples were as follows: alpha (351, 46.184%), exocrine (185, 24.342%), beta (79, 10.395%), delta (63, 8.289%), ductal (38, 5%), macrophage (22, 2.895%), mast (4, 0.526%), endothelial (3, 0.395%), gamma (3, 0.395%), and stellate (1, 0.132%).

### 3.13 Cell–cell communication and pseudotime trajectory analysis

**Figure 14:**
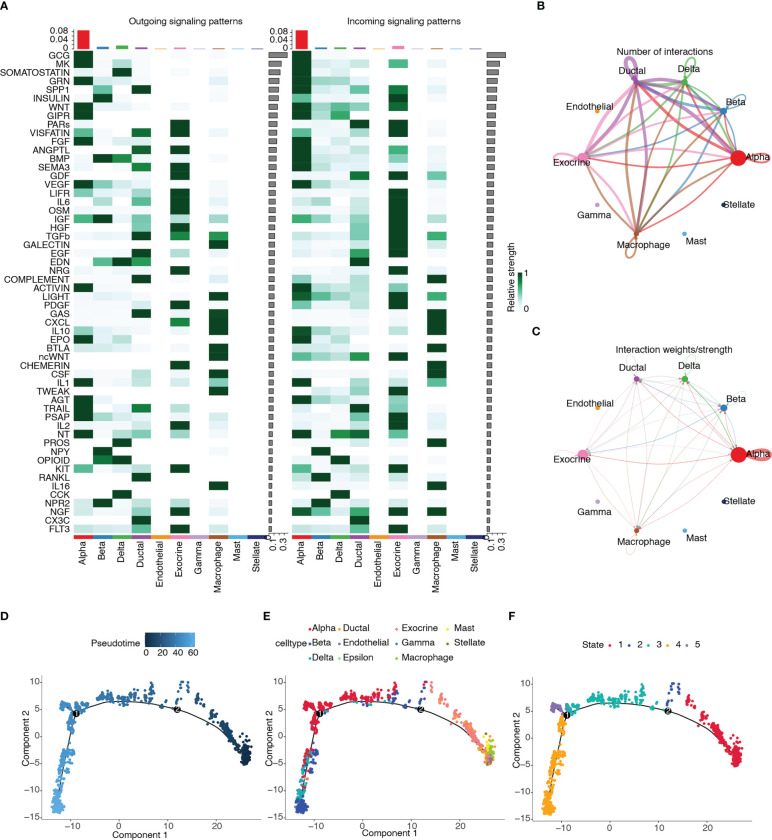
Cell–cell communication and pseudotime trajectory analysis. **(A)** Heatmap of the contribution of outgoing and incoming pathways. **(B)** Circle plot of numbers of cell interactions. **(C)** Circle plot of weights of cell interactions. **(D)** Pseudotime trajectory colored according to pseudotime progression. **(E)** Pseudotime trajectory colored according to cell type. **(F)** Pseudotime trajectory colored according to state of cell population.

CellChat was used to infer and quantify intercellular communication. **Figure 14A** shows the contribution of the outgoing and incoming pathways to cell types. The outgoing and incoming pathways with the largest contribution were *GCG*, and the cell type with the strongest correlation was alpha. We then drew circle plots to visualize the numbers (**Figure 14B**) and weights (**Figure 14C**) of cell interactions and found that alpha cells were the largest in number and weight.

Pseudotime trajectory analysis was performed on the cell types of the T2D samples by using the Monocle package. The trajectory plots of cells are colored according to pseudotime progression, cell type, and state of cell population (**Figures 14D–F**). **Figure 14E** shows that the trajectory plot was divided into five pseudotemporal states. Alpha cells were mainly distributed in states 3, 4, and 5.

### 3.14 GSEA among different clusters

**Figure 15:**
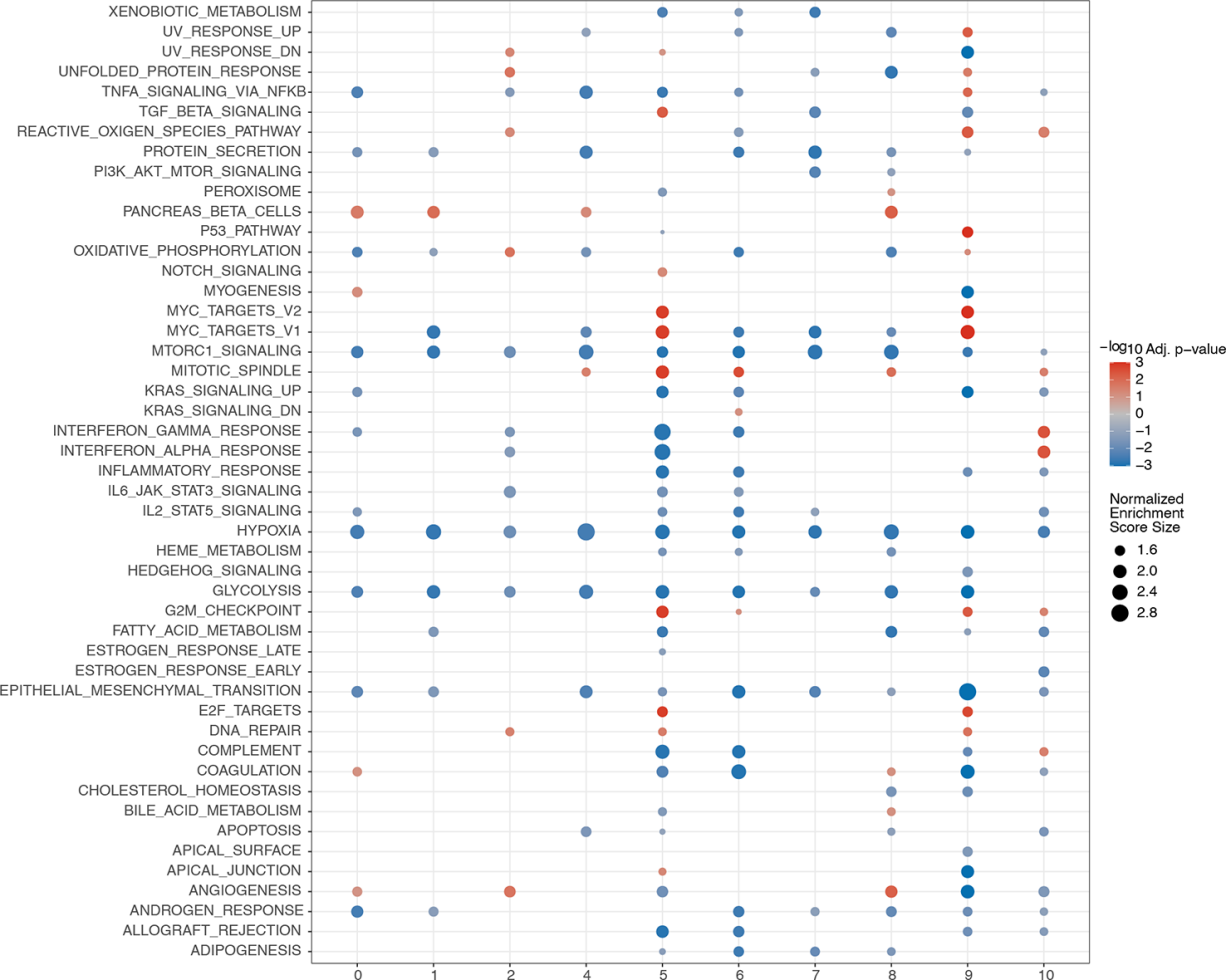
GSEA analyses among different clusters. The abscissa represents the cell clusters, and the ordinate denotes rich pathway. Circle size reflects the normalized enrichment score. Red represents upregulation, while blue represents downregulation.

GSEA was performed among the 11 clusters to illustrate the biological functions associated with these clusters (**Figure 15**). A relatively large number of differential pathways were enriched in clusters 5(endothelial cells) and 9 (macrophages). In addition, the TGF, myc targets v1, myc targets v2, mitotic spindle, G2M checkpoint, and E2F target signaling pathways were significantly upregulated in cluster 5. Results showed that UV response upregulation, unfolded protein response, tumor necrosis factor α signaling *via* the NF-κB, reactive oxygen species, P53, myc targets v2, myc targets v1, G2M checkpoint, E2F targets, and DNA repair signaling pathways were significantly upregulated in cluster 9.

## 4 Discussion

T2D is a lifelong metabolic disorder with a worldwide prevalence of 10.5% in 2021 (59). T2D is a genetic disease, but its genetics remains poorly understood (3). Therefore, the pathophysiological mechanisms that trigger T2D should be elucidated to improve the management of this disease. In this study, we explored the potential crucial genes and pathways associated with pyroptosis and immune infiltration in T2D in a merged matrix from three bulk RNA-seq datasets of islets. We constructed miRNA and transcriptional networks based on these hub genes and performed functional analyses. Furthermore, these pyroptosis-, immune-, and T2D-related genes were analyzed using scRNA-seq data to explain the cellular heterogeneity in T2D. Our study provides insights into the molecular mechanisms underlying islet inflammation and human T2D pathogenesis caused by islet dysfunction.

In this study, we identified 918 T2D-related DEGs in the merged matrix. GO analysis revealed that these genes were highly enriched for the synthesis, secretion, and mode of action of hormones or peptides. The immune scores were significantly higher in the T2D group than in the non-diabetic group. Then, 550 immune- and T2D-related DEGs were obtained. Our results indicated that the immune system plays a crucial role in T2D pathogenesis, consistent with previous findings that immunologic–metabolic crosstalk is involved in T2D development (60–62). Accumulating evidence has shown that pyroptosis, a programmed proinflammatory cell death pathway, is activated during T2D development (63–65). We identified 31 pyroptosis-related genes in the merged matrix.

After LASSO regression, *GBP2*, *NLRP1*, and *NOD2* were used to construct a clinical prediction model. The AUC value of the model was 0.968, suggesting that this model exhibited excellent accuracy (66) and might be an ideal target for the diagnosis of T2D. *GBP2*, a member of the GTPase family, triggers pyroptosis by supporting inflammasome activation (67). *GBP2* has been identified as a candidate gene in diabetic retinopathy (68). Polymorphisms in *NLRP1* affect susceptibility to type 1 diabetes in the Chinese Han population (69). *NOD2*, a member of the nucleotide oligomerization domain (NOD)-linked receptor family, is associated with immune and chronic inflammatory disorders (70). *NOD2* is upregulated in diabetic cardiomyopathy and silencing this gene could protect against diabetes-induced cardiomyopathy (71). However, Ozbayer et al. reported that *NOD2* is not associated with T2D (72).

We identified 115 genes associated with pyroptosis, immune cell infiltration, and T2D and considered 17 of these genes to be pyroptosis-related hub genes. In the present study, *INS* was the top gene with the most nodes, and it appeared to be closely related to T2D. Mutations and translation defects in *INS* have been associated with diabetes (73). A recent study has reported that *INS* could be regulated by m6A modification, providing a prediction for the occurrence of T2D (74). Most of the other hub genes, including *GCG* (75), *NEUROD1* (76), *PCSK1* (77), *ABCC8* (78), *STMN2* (79), *IAPP* (80), *KCNQ1* (81), *NKX2-2* (82), *SCG5* (83), *CPE*, and *GNAS* (84), have been strongly associated with the onset or development of T2D. *CHGA*, *GAD2*, *GRIA2*, *CHGB*, and *SLC17A6* have not been previously reported to be associated with T2D.

To probe the potential upstream and downstream regulators of hub genes, we constructed miRNA–mRNA interaction and transcriptional regulatory networks. Each miRNA can target many mRNAs, and a single mRNA can be regulated by several miRNAs. Growing evidence has indicated that miRNAs, endogenous regulators of gene expression, are involved in T2D pathogenesis (85). Srividya et al. have summarized miRNAs as biomarkers for T2D diagnosis (86). A meta-analysis identified 40 miRNAs that are associated with T2D (87). Transcriptional regulatory networks describe the regulatory interactions between TFs and their target genes. Similar to miRNAs, a single TF usually regulates multiple genes, and a gene is regulated by multiple TFs. In the present study, GO analysis revealed that these genes were also highly enriched for the synthesis, secretion, and mode of action of hormones or peptides. These results were consistent with the GO analysis results of the T2D-related DEGs. For KEGG pathway analysis, the pyroptosis-related hub genes were enriched in maturity-onset diabetes of the young, insulin secretion, type 1 diabetes mellitus, and glutamatergic synapses. The signaling pathway of maturity-onset diabetes of the young showed the greatest impact on T2D. Previous studies have shown that the maturity-onset diabetes of the young pathway plays a significant role in T2D pathogenesis (88, 89). GSEA and GSVA were performed for the high- and low-pyroptosis score groups, respectively. These results complemented the GO and KEGG pathway analyses.

Abnormal differentiation of components of the immune system is involved in the progression of T2D (90–93). In the present study, disease states were almost uniformly distributed among pyroptosis states and among the wound healing (C1), inflammatory (C3), and lymphocyte-depleted (C4) immune subtypes. We determined the abundance of 13 immune cell types in the merged matrix and found correlations among them. Multiple immune cell types were identified in the islets. Immune cells and inflammatory mediators accumulate in the islets of both animal models and humans (7, 17, 94). A recent study has confirmed the effect of T cells on T2D (95). Wang et al. summarized the role of the imbalance between T helper 17 and regulatory T cells in T2D (96). In the present study, we identified two molecular subtypes (clusters 1 and 2) by performing unsupervised clustering for T2D samples in the merged matrix. Cluster 2 showed a high expression of 17 pyroptosis-related hub genes and a high abundance two types of immune cells (monocytes and CD8+ T cells). Macrophage count increases in the islets of patients with T2D, and islet macrophage infiltration correlates with islet dysfunction (7, 8). Inflammation triggers the differentiation of monocytes into macrophages. Wu et al. performed an analysis of single-cell data on human pancreas and found that monocytes and CD8+ T cells are enriched in the T2D pancreas (97). Our results are consistent with previous reports that monocytes and macrophages are the primary immune cell subsets that contribute to islet inflammation during T2D development (98). We also found correlations between the 17 pyroptosis-related hub genes and 13 immune cell types. The relationship between pyroptosis regulators and immune infiltrate characterization has been discussed in diseases, including cancer and periodontitis (99, 100).

In this study, we identified 11 cell types from the scRNA-seq dataset. The islets of Langerhans are composed of multiple types of endocrine cells (alpha, beta, delta, gamma, and epsilon) with distinct functions and non-endocrine cells (101–103). Maayan et al. found that human pancreatic cells can be divided into 14 cell populations based on the expression of unique transcripts and references (104). Joshua et al. performed single-nucleus ATAC-seq on human pancreatic islets and identified 12 distinct cell clusters (105). The location of the respective marker genes was consistent with the distribution of each cluster, suggesting the accuracy of the cluster analysis and major cell-type identification. We found differences in the distribution of various cell types between the T2D and non-T2D samples, revealing the heterogeneity caused by T2D. Our results are in concordance with the results of a previous study that β-cell mass decreases and α-cell volume increases in the pancreatic tissue of patients with T2D (106). We obtained candidate hub genes for different cell subtypes by intersecting DEGs with 115 genes. *INS* and *IAPP* were determined to be pyroptosis-related and candidate hub genes. The relationships of *INS* and *IAPP* with T2D have been reported in previous studies (74, 80, 107).

Our intercellular communication analysis showed that the gene with the largest contribution was *GCG* and the cell type with the strongest correlation with T2D was alpha. *GCG* has been considered an islet alpha cell type-specific gene to cluster alpha cells (56, 108). *GCG* encodes a variety of peptides, of which glucagon and glucagon-like peptide-1 have attracted increasing attention because of their effects on glucose metabolism. In the last few decades, multiple novel drugs for T2D treatment have been developed based on the utilization of the signaling systems of *GCG* products (109). Pseudotime trajectory analysis showed that cell types of T2D existed along the trajectory, and alpha cells were located at the end of the trajectory line. Chiou et al. presented a detailed characterization of islet cell types and state regulatory programs, which provided a wide perspective to interpret the genetic mechanisms underlying T2D (104). In the present study, we found that myc targets v1, myc targets v2, G2M checkpoint, and E2F target pathways were significantly upregulated in clusters 5 and 9. MYC is a signaling pathway capable of regulating apoptotic cell death, proliferation, survival, and differentiation (110). A previous study found that myc, a member of the Wnt signaling pathway, is upregulated in the islets of patients with T2D (111). The G2M cell cycle checkpoint plays a critical role in diabetic oxidative stress signaling (112). The E2F signaling pathway is associated with the proliferation and regeneration of islets from patients with T2D (113). These results suggest that cell death, proliferation, and regeneration play important roles in islet dysfunction in T2D.

However, this study has some limitations. First, the merged and scRNA-seq datasets used in this study are still relatively small. A larger number of cells in the scRNA-seq dataset are required to identify rare cell subpopulations or detect minor changes in gene expression. Second, the lack of detailed clinical data hindered the evaluation of the relationship between clinical characteristics of T2D and gene expression. Third, further experiments, such as quantitative real-time PCR, western blot, and immunohistochemistry, are warranted to clarify the functions of the hub genes in T2D.

In conclusion, we identified candidate genes associated with pyroptosis, immune infiltration, and disease phenotypes in T2D development. Furthermore, we presented a detailed characterization of islet cell types and their expression patterns in T2D. The combined bulk RNA-seq data and cell type-specific data of islets provided insights into the molecular mechanisms underlying T2D and novel therapeutic targets for T2D treatment. We believe this hypothesis generating study provides a critical resource for understanding of islet dysfunction and T2D pathogenesis.

## Data availability statement

The datasets presented in this study can be found in online repositories. The names of the repository/repositories and accession number(s) can be found within the article/**Supplementary Materials**.

## Author contributions

YS and CH conceptualized the study and analyzed the data. YS drafted the manuscript. YS, CH, and YJ drew the figures. MY and ZX performed the data acquisition and collation. YS, CH, YJ, MY, ZX, LY, WZ, and YX collaborated with the interpretation and discussion of the results. YX critically revised the manuscript. All authors contributed to the article and approved the submitted version.
