# Supplementary figures and images for: Bulk and single-cell transcriptome analyses of islet tissue unravel gene signatures associated with pyroptosis and immune infiltration in type 2 diabetes
Source: Front Endocrinol (Lausanne). 2023 Mar 9;14:1132194. doi: 10.3389/fendo.2023.1132194 (PMC10034023; doi:10.3389/fendo.2023.1132194)

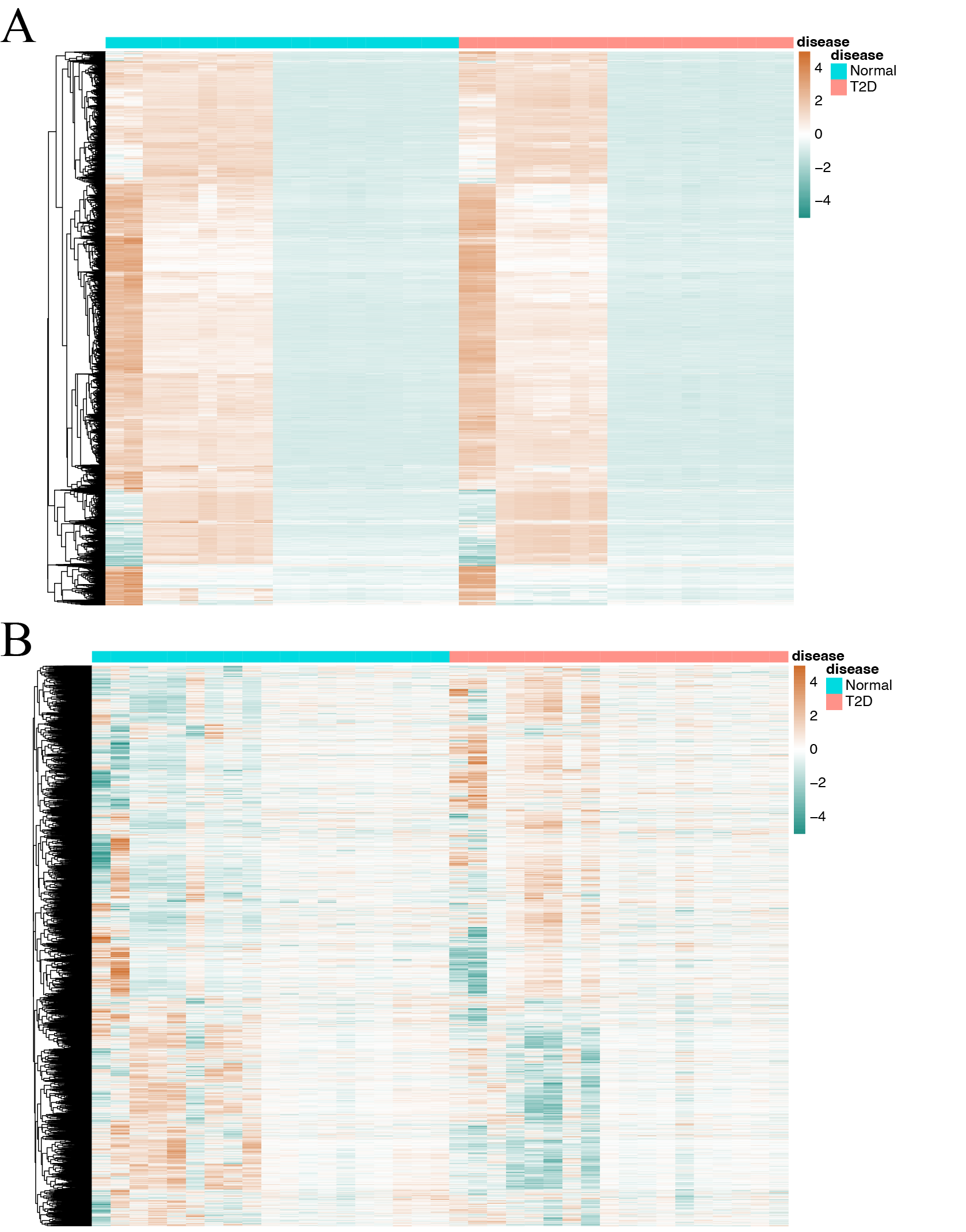

Supplement: Supplementary file 1 [file Image_1.tif]

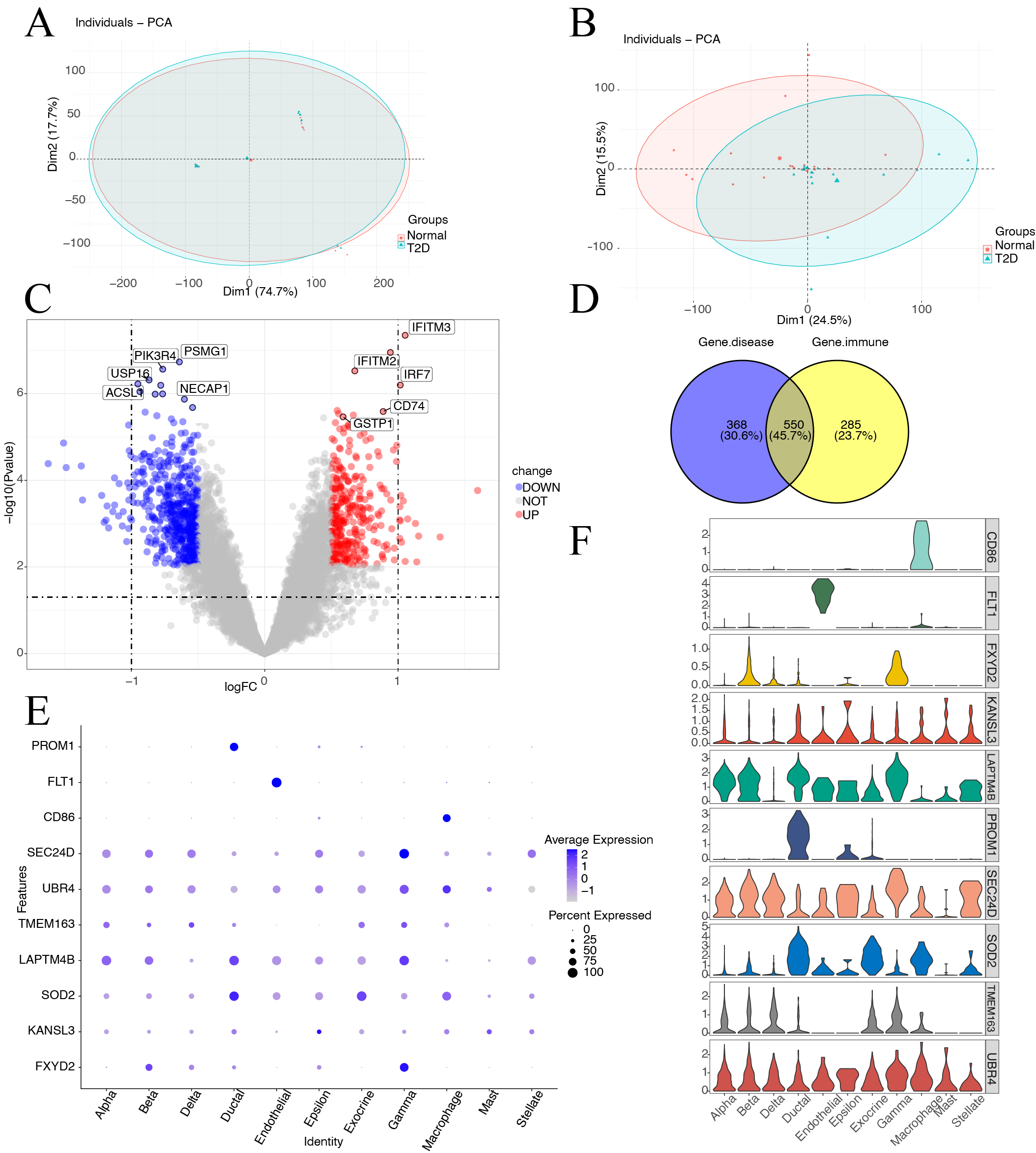

Supplement: Supplementary file 2 [file Image_2.tif]

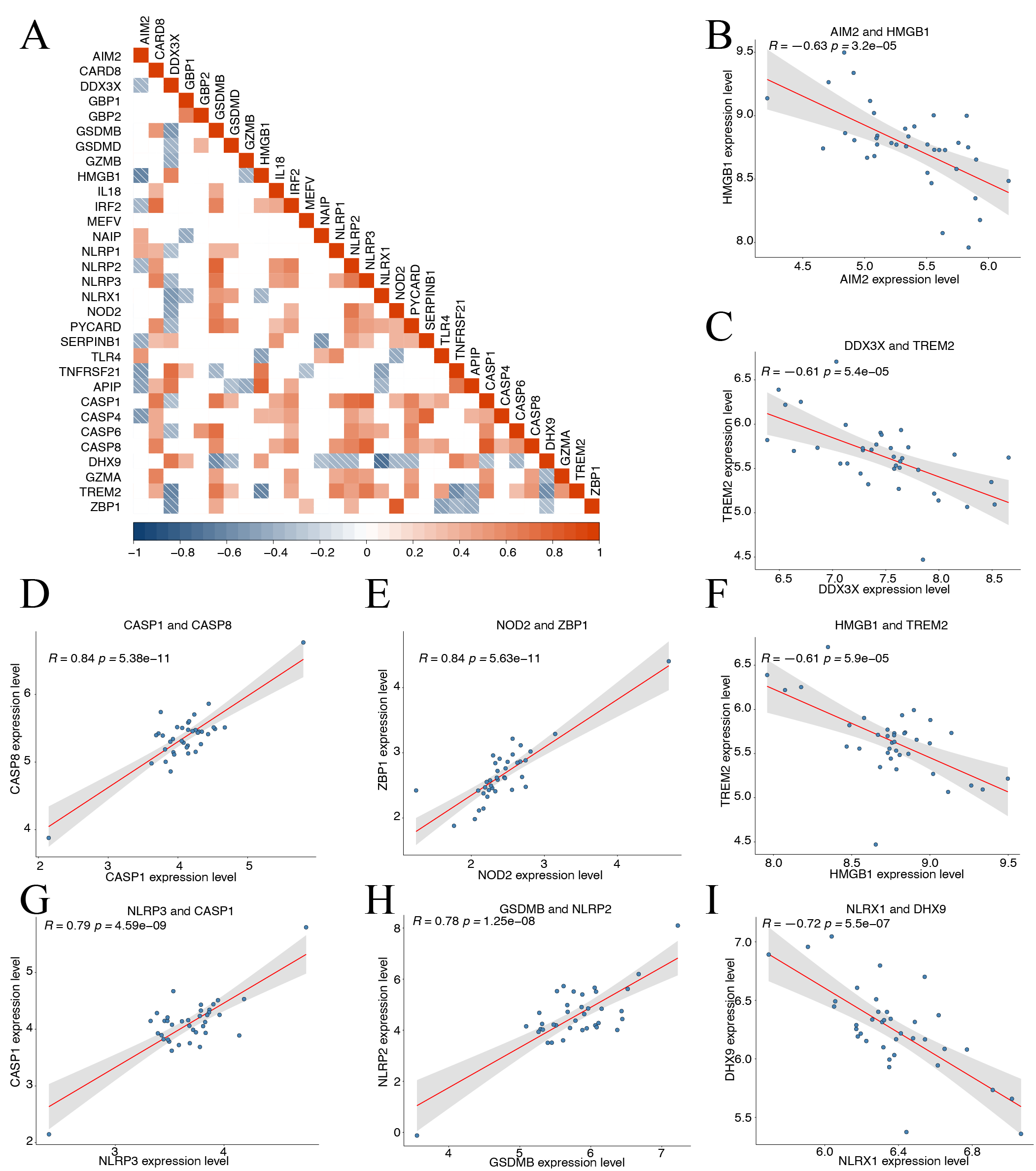

Supplement: Supplementary file 3 [file Image_3.tif]

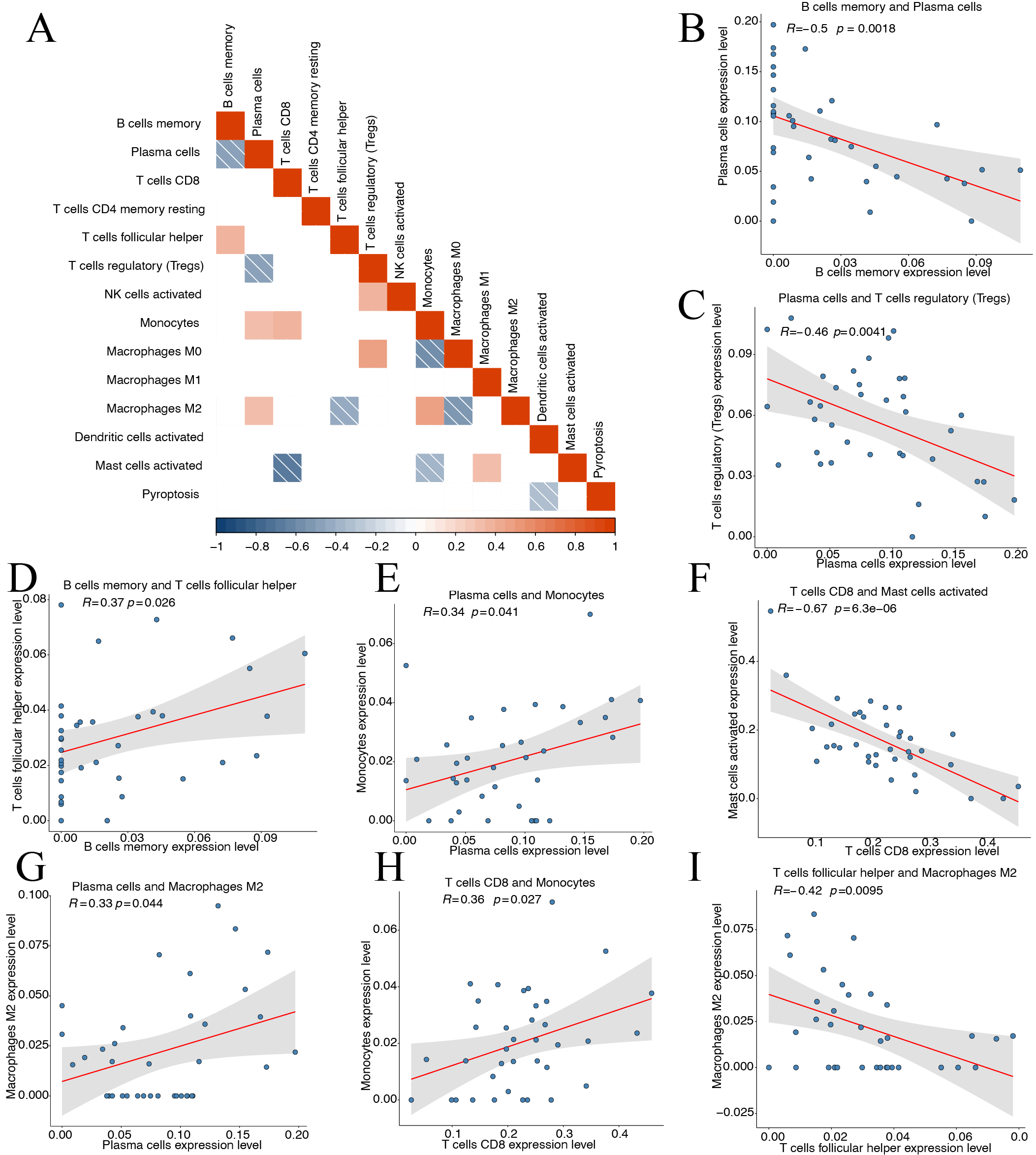

Supplement: Supplementary file 4 [file Image_4.tif]

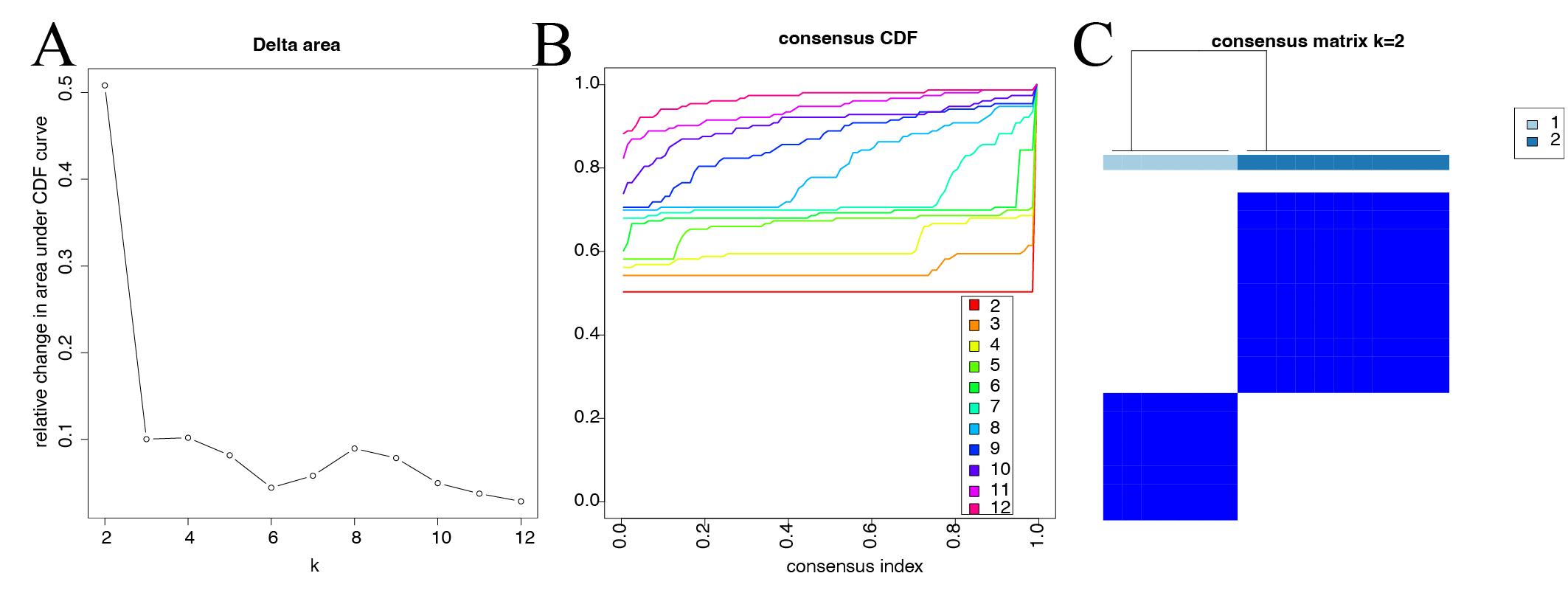

Supplement: Supplementary file 5 [file Image_5.tif]

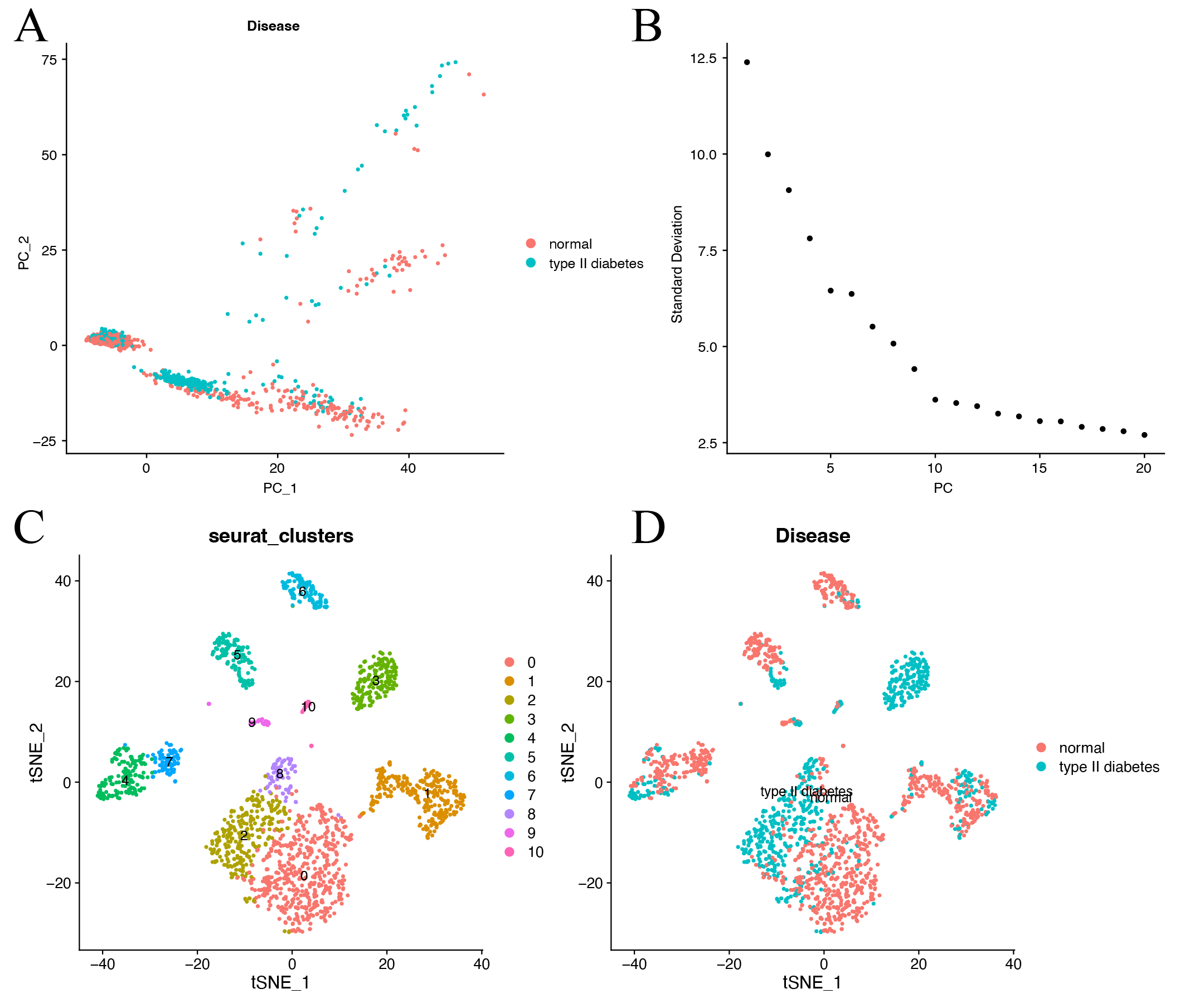

Supplement: Supplementary file 6 [file Image_6.tif]
