# Supplementary material for: Bulk and single-cell transcriptome analyses of islet tissue unravel gene signatures associated with pyroptosis and immune infiltration in type 2 diabetes
Source: Front Endocrinol (Lausanne). 2023 Mar 9;14:1132194. doi: 10.3389/fendo.2023.1132194 (PMC10034023; doi:10.3389/fendo.2023.1132194)
Supplement: Supplementary file 13 [file Presentation_1.pdf]

Supplementary Material

Bulk and Single-cell Transcriptome Analyses of Islet Tissue Unravel Gene Signatures Associated with Pyroptosis and Immune Infiltration in Type 2 Diabetes

Yaxian Song, Chen He, Yushan Xu*

*** Correspondence:** Yushan Xu: xuyushan@kmmu.edu.cn

1 Supplementary Figures and Tables

1.1 Supplementary Figures

Figure S1 Heat maps for a merged dataset before and after batch effect correction. (**A**) Heat map of a merged dataset without batch effect correction. (**B**) Heat map of a merged dataset after batch effect correction.

Figure S2 PCA plots before and after batch effect correction; Immune and T2D-related genes in the merged matrix；Expression levels of marker genes in single-cell expression data. (**A**) PCA of a merged dataset without batch effect correction. (**B**) PCA of a merged dataset after batch effect correction. (**C**) Volcano plot of 835 immune-related genes between the high- and low-immune score group. (**D**) Venn diagram of 835 immune-related DEGs and 918 T2D-related DEGs. (**E**) Bubble plot showing expression levels of marker genes in 11 cell types. (**F**) Violin plot showing expression levels of marker genes in 11 cell types.

Figure S3 Correlations analyses of pyroptosis-related genes in T2D. (**A**) Correlation heatmap showing the correlated expression patterns of 31 pyroptosis-related genes. (**B–I**) Top four significant negative and positive gene–gene correlations are shown by correlation scatter plots.

Figure S4 Correlation analyses among infiltrating immune cells. (**A**) Correlation heatmap showing correlations among the 13 types of immune cells. (**B**) Correlation of memory B cells and plasma cells ($r = -0.5$, $P = 0.0018$). (**C**) The correlation of plasma cells and regulatory T cells (Tregs) ($r = -0.461$, $P = 0.0041$). (**D**) Correlation of memory B cells and follicular helper T cells ($r = 0.367$, $P = 0.0026$). (**E**) Correlation of plasma cells and monocytes ($r = 0.337$, $P < 0.05$). (**F**) Correlation of CD8+ T cells and activated mast cells ($r = -0.668$, $P < 0.001$). (**G**) Correlation of plasma cells and M2 macrophages ($r = 0.334$, $P = 0.044$). (**H**) Correlation of CD8+ T cells and monocytes ($r = 0.363$, $P = 0.027$). (**I**) Correlation of follicular helper T cells and M2 macrophages ($r = -0.421$, $P = 0.0095$).

Figure S5 Unsupervised clustering of T2D samples. (**A**) Delta area curve. (**B**) Consensus cumulative distribution functions (CDF). (**C**) Consensus matrix heatmap. Values of consensus in the consensus matrix ranged from zero (white) to one (dark blue): zero means samples “never cluster together”; one means samples “always cluster together.”

Figure S6 Principal component analysis plot and graph-based clustering of the GSE153855 dataset. (**A**) Overdistribution of the data is displayed by plotting principal component 1 (PC1) versus principal component 2 (PC2). (**B**) Plot showing relationships between the number of principal components and the standard deviation. (**C**) Dimensionality reduction plot using t-SNE showing 11 distinct clusters for T2D samples. (**D**) Dimensionality reduction plot using t-SNE showing clusters for T2D and non-T2D samples.

1.2 Supplementary Tables

Table 1 31 Pyroptosis-related genes expressed in the merged matrix.

Table 2 GO analysis of T2D-related genes in the merged matrix

Table 3 550 immune and T2D-related genes in the merged matrix

Table 4 115 genes associated with pyroptosis, immune cell infiltration, and T2D in the merged matrix

Table 5 GSEA enrichment analyses between the high- and low-pyroptosis score groups.

Table 6 GSVA enrichment analyses between the high- and low-pyroptosis score groups.
